# LTR-retrotransposon transcriptome modulation in response to endotoxin-induced stress in PBMCs

**DOI:** 10.1186/s12864-018-4901-9

**Published:** 2018-07-05

**Authors:** Marine Mommert, Olivier Tabone, Guy Oriol, Elisabeth Cerrato, Audrey Guichard, Magali Naville, Paola Fournier, Jean-Nicolas Volff, Alexandre Pachot, Guillaume Monneret, Fabienne Venet, Karen Brengel-Pesce, Julien Textoris, François Mallet

**Affiliations:** 1 Joint research unit, Hospice Civils de Lyon, bioMerieux, Centre Hospitalier Lyon Sud, 165 Chemin du Grand Revoyet, 69310 Pierre-Benite, France; 2 EA 7426 Pathophysiology of Injury-induced Immunosuppression, University of Lyon1-Hospices Civils de Lyon-bioMérieux, Hôpital Edouard Herriot, 5 Place d’Arsonval, 69437 Lyon, Cedex 3 France; 3 Institut de Genomique Fonctionnelle de Lyon, Univ Lyon, CNRS UMR 5242, Ecole Normale Superieure de Lyon, Universite Claude Bernard Lyon, 1, 46 allee d’Italie, F-69364 Lyon, France; 4 Hospices Civils de Lyon, Immunology Laboratory, Groupement Hospitalier Edouard Herriot, Lyon, France; 5 Hospices Civils de Lyon, Department of Anaesthesiology and Critical Care Medicine, Groupement Hospitalier Edouard Herriot, Université Claude Bernard Lyon 1, Lyon, France

**Keywords:** HERV transcriptome, PBMCs, LPS, Endotoxin tolerance, Signalling pathways, Sepsis

## Abstract

**Background:**

Human Endogenous Retroviruses (HERVs) and Mammalian apparent LTR-retrotransposons (MaLRs) represent the 8% of our genome and are distributed among our 46 chromosomes. These LTR-retrotransposons are thought to be essentially silent except in cancer, autoimmunity and placental development. Their Long Terminal Repeats (LTRs) constitute putative promoter or polyA regulatory sequences. In this study, we used a recently described high-density microarray which can be used to study HERV/MaLR transcriptome including 353,994 HERV/MaLR loci and 1559 immunity-related genes.

**Results:**

We described, for the first time, the HERV transcriptome in peripheral blood mononuclear cells (PBMCs) using a cellular model mimicking inflammatory response and monocyte anergy observed after septic shock. About 5.6% of the HERV/MaLR repertoire is transcribed in PBMCs. Roughly one-tenth [5.7–13.1%] of LTRs exhibit a putative constitutive promoter or polyA function while one-quarter [19.5–27.6%] may shift from silent to active. Evidence was given that some HERVs/MaLRs and genes may share similar regulation control under lipopolysaccharide (LPS) stimulation conditions. Stimulus-dependent response confirms that HERV expression is tightly regulated in PBMCs. Altogether, these observations make it possible to integrate 62 HERVs/MaLRs and 26 genes in 11 canonical pathways and suggest a link between HERV expression and immune response. The transcriptional modulation of HERVs located close to genes such as OAS2/3 and IFI44/IFI44L or at a great distance from genes was discussed.

**Conclusion:**

This microarray-based approach revealed the expression of about 47,466 distinct HERV loci and identified 951 putative promoter LTRs and 744 putative polyA LTRs in PBMCs. HERV/MaLR expression was shown to be tightly modulated under several stimuli including high-dose and low-dose LPS and Interferon-γ (IFN-γ). HERV incorporation at the crossroads of immune response pathways paves the way for further functional studies and analyses of the HERV transcriptome in altered immune responses in vivo such as in sepsis.

**Electronic supplementary material:**

The online version of this article (10.1186/s12864-018-4901-9) contains supplementary material, which is available to authorized users.

## Background

Retrovirus-like sequences represent the 8% of the human genome [1]. They consist of some 200,000 Human Endogenous Retroviruses (HERVs) and 240,000 Mammalian Apparent LTR retrotransposons (MaLRs). HERVs are remnants of ancestral and independent retroviral infections within the germ line. The parental integrated retroviral DNA or provirus is flanked by two Long Terminal Repeats (LTR). The 5’LTR contains the promoter and enhancer signals initiating transcription, while the 3’LTR contains the polyadenylation signal terminating transcription. Between the two LTRs lie at least three genes coding for the structural proteins (*gag*), the enzymatic proteins (*pro-pol*), and the envelope glycoprotein (*env*). MaLR structure is similar except for the absence of an *env* gene. Since endogenization, proviruses have propagated all along the genome by reinfection and retrotransposition events. Due to the general absence of selection pressure, most of the elements contain substitutions, insertions and deletions. However, a few hundred large open reading frames (ORF) remain [2], including *env* ORFs, of which Syncytins support essential functions in placental development [3]. In addition, numerous HERV elements consist of solitary LTRs, resulting from the loss of coding genes by recombination between the two flanking LTRs. All these mechanisms lead to complex multicopy groups, (reviewed in [4–6]). Each group consists of heterogeneous elements, all defective for replication, and thus engaged in a vertical mode of transmission exclusively. While bioinformatics approaches have identified 103 HERV groups and 1 MaLR group [1], only 40 HERV groups have been characterised in wet-lab studies [7–9]. Based on the homology of *pol* sequences between HERVs and exogenous retroviruses, these well-defined groups can be classified as gamma-, beta-, spuma- and epsilon- retroviruses [10].

As repeated elements and due to their organisation into groups, (H)ERVs may be involved in genomic plasticity during evolution, being preferential recombination sites within or between chromosomes [11]. Under physiological conditions, HERV elements are subject to strong epigenetic controls either in terms of methylation or histone code [12]. Nevertheless, in various diverse situations, HERV elements have been shown to be transcribed. HERV transcription has been observed in organ-specific (e.g. brain for multiple sclerosis) and systemic (e.g. lupus erythematosus) autoimmune diseases, and HERV-driven mechanisms involving molecular mimicry and immune dysregulation have been proposed [13–15]. HERV expression has also been researched in cancer with regard to the oncogenic properties of infectious retroviruses and epigenetic changes observed in cancer [12, 16]. The latter highlighted LTR-driven transactivation of cellular proto-oncogenes or the expression of HERV-encoded *Env,* NP9 or Rec candidate oncogenes. HERVs also contribute to the physiopathology of their host at multiple levels. In brief, (i) solo or proviral LTR can modulate the expression of adjacent cellular genes, in addition to their autonomous function in controlling retroviral expression [17–20], (ii) the expression of HERV proteins with conventional retroviral functions can influence the host’s physiological or pathological states, like fusion for Syncytin-1 [21], immunomodulation for Env HERV-H and Syncytin-2 [22, 23], RNA nuclear export for Rec [24], and even viral-like particle formation derived from HML-2 [25], and finally (iii) non-coding HERV-expressed sequences may also be biologically active, e.g. HERV-H loci involved in the maintenance of pluripotency in human cells [26]. In all cases, deciphering an HERV biological function starts with the analysis of its expression, which is far from being simple due to both complex biological mechanisms and technical challenges. Such biological complexity is illustrated by one extensively described HERV-W element, the ERVWE1 locus, encoding placental fusogenic Syncytin-1 Env. ERVWE1 expression is driven by its own 5’LTR U3 promoter and adjacent MaLR LTR enhancer. Restricted expression outside the trophoblast is controlled at the LTRslevel by CpG methylation and/or repressive histone mark H3K9me3, and at the splicing level, at least in part, by H3K36me3 along the intron–exon boundary (reviewed in [3]). Experimentally, the challenge of the individual identification of transcriptionally active HERV loci was recently addressed using NGS [27] and high-density microarray [28–30] technologies. Indeed, first [28], second [29] and third [30] generations of custom HERV-dedicated microarrays aimed to solve the antagonism between the specificity of individual locus recognition and exhaustiveness of the HERVome. Although addressing a limited number of groups, the first two generations of HERV-dedicated microarrays confirmed that reproductive organs and solid tumours are major sites of HERV expression, and highlighted the tissue specificity/tropism of expressed HERV elements [28, 29].

Most of the literature concerning HERVs focuses on autoimmune diseases, cancers and placental physiopathology, all these contexts being associated with local or systemic modulation of the immune response. Indeed, there is a growing line of evidence that HERVs may directly shape and regulate our immune system [31–33]. The first evidence of the expression of HERVs beta-retroviruses in PBMCs was reported in healthy volunteers 20 years ago, using *pol*-based pan retroviral PCR [34], following Northern-blot-based seminal scrutiny [35]. This observation was extended to the HERV-H and HERV-W gamma-retrovirus groups using similar PCR-based technology 20 years later [36]. It was demonstrated that the level of HERV expression in the PBMC compartment is modulated in solid organ cancers, autoimmune diseases, infectious diseases, as well as immunocompromised states, e.g. HML-2 in prostate cancer [37], HERV-W/MSRV in multiple sclerosis [38], HERV-W in EBV-infected multiple sclerosis patients [39], and HML-2 in HIV infected patients [40]. In line with this, HERV expression in PBMCs is modulated by microbial components, the differentiation state of the cell and cytokines. Bacteria-derived components such as LPS increase HERV-W, HERV-K, HERV-H, and decrease HERV-E group expression in monocyte-derived macrophage (MDM) cell lines [41]. Recently, taking advantage of probes targeting HERVs in commercial microarrays, the immune cell activation by microbial signals in vitro induces global modulation of endogenous retroelements [42]. HERV-W, HERV-K and HERV-H RNA levels are increased during monocyte differentiation [41]. HERV expression is modulated by cytokines, as observed for ERV3 in the U-937 monocytic cell line [43], or also for MSRV released from PBMCs stimulated by TNF-α and IFN-γ and inhibited by IFN-α [44]. Moreover, Env of HERV-W and HERV-H groups are expressed on the surface of B cells and monocytes in patients with active multiple sclerosis [45]. Syncytin-1 has also been observed in other pro-inflammatory states in skin-homing non-recirculating mycosis fungoides T cells [46]. Conversely, immunosuppressive properties of Syncytin-1 [47, 48] and Env HERV-H [22] have been described in different contexts [49]. These data support the hypothesis that HERVs are expressed in inflammatory and immunosuppressive contexts and have direct or indirect interactions with the immune host response.

Thus, the considerable improvement inthe molecular and cellular biology tools helps demonstrate that many HERVs are not silent in many contexts. The use ofunique locus candidate approaches helps understand their role in pathophysiological phenomena. We recently introduced a third generation of HERV-dedicated microarray, in high-density Affymetrix format, which makes it possible to measure HERVs at the individual locus level. It targets almost complete coverage of HERVs and MaLRs LTR-retrotransposons of the GRCh38 version of the human genome and Dfam 1.1 database of repetitive DNA element sequence alignments. The chip also targets more than 1500 human genes mainly coding for immunity-related proteins [30]. We investigated HERVs and MaLRs expression in PBMCs by modelling monocyte endotoxin tolerance, consisting in low-dose LPS priming of PBMCs and mimicking monocyte anergy observed in sepsis patients [50–52]. Symmetrically, PBMCs were stimulated by single high-dose LPS mimicking gram-negative bacterial infection and illustrating an inflammatory context. This article i) provides an overview of the HERV transcriptome in PBMCs associated with functional LTR characterisation; ii) shows the stimulus-dependent co-modulation of HERVs/MaLRs and genes, including tolerisable reversible phenotypes; and iii) demonstrates an integrative view of HERVs/MaLRs and genes in canonical immunity pathways, illustrating the multifaceted nature of interactions between LTR retrotransposons and genes.

## Results

### Detection of the HERV transcriptome in PBMCs

**Fig. 1:**
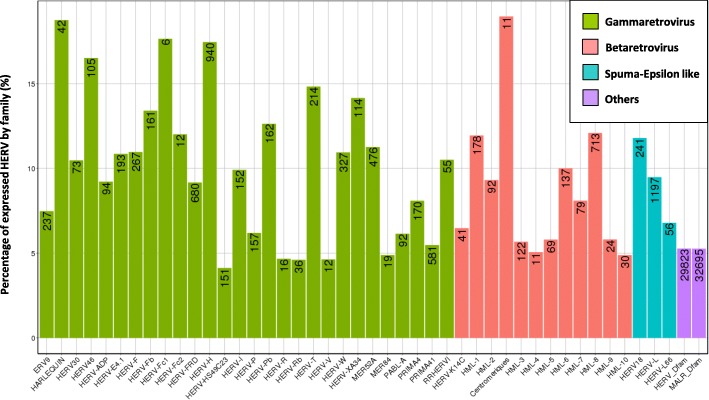
The HERV transcriptome in PBMCs. Percentages and absolute counts of positive signal-associated probesets within individual groups of the “HERV_prototypes” repertoire and HERV_Dfam and MaLR_Dfam repertoires. A probeset was included as reflecting a significant transcriptional activity if its normalised intensity was over an intensity threshold of 2^5.5^ in at least 14 out of the 45 samples (for all conditions). This conservatory threshold was defined as the minimal intensity level shared by all repertoires that exhibited an acceptable variability, i.e. the 75th percentile of the distribution of the variation coefficient as a function of intensity should be lower than 10% (illustrated in Additional file 1: Figure S1). HERV prototype groups were grouped by retrovirus classes, namely gammaretrovirus (green), betaretrovirus (red) and spuma-epsilon like retrovirus (blue). The HERV and MaLR Dfam repertoires were each depicted as a global homogeneous entity (purple)

In order to present an overview of the HERV/MaLR transcriptome in PBMCs, we used various LPS challenges to mimic healthy, inflammatory and immunocompromised states using the previously described endotoxin tolerance model [50]. The transcriptome was scrutinised using a custom Affymetrix HERV-V3 microarray [30] which can discriminate 174,852 HERV elements, 179,142 MaLR elements and a set of 1559 genes. In addition to the gene probesets designed in U133plus2 and HTA Affymetrix formats, the chip contains a set of highly informative probesets corresponding to accurately annotated HERV loci hereinafter referred to as “HERV_prototypes”, and two sets of probesets corresponding to roughly annotated HERVs/MaLRs elements called hereafter “HERV_Dfam” and “MaLR_Dfam” (summarised in Additional file 2: Table S1). In brief, the “HERV prototypes” repertoire was retrieved from selected prototype loci. These are sets of loci which maintain the largest open reading frames for *gag pol env* genes within a proviral structure flanked by two complete LTR sequences. From these elements, a repeatmasker-based alignment procedure retrieved 29,271 loci divided into 42 groups. The “HERV_Dfam” and “MaLR_Dfam” repertoires were retrieved from Dfam, a database of repetitive elements detected by RepBase consensus and based on Hidden Markov Models (HMM) [53] (“Proto versus Dfam” tab, Additional file 2: Table S1). Redundancy between repertoires was removed from the HERV/MaLR repertoire. A summary of the absolute counts and the relative abundance of transcriptionally active elements of the HERV/MaLR transcriptome is given in Fig. 1, at the probeset level. Notably, although the hybridisation assessment quality was equivalent across repeated elements and gene repertoires [30], a higher proportion of gene probesets were transcriptionally active, i.e. 52% (42,560 probesets). Overall, 5.6% of targeted HERVs/MaLRs (71,063 probesets) were transcriptionally active in PBMCs. More precisely, 9.4% of the well-described “HERV_prototypes” repertoire, and 5.5% of HERV_Dfam and MaLR_Dfam were expressed. Among the 9.4% expressed prototype elements, 6.1, 1.7 and 1.6% belonged to gamma-, beta- and spuma/epsilon-like retrovirus classes, respectively. On moving from classes towards groups, all well-defined HERVs groups had expressed loci in PBMCs. Notably, within gamma-retroviruses, the largest HERV-H group is that with the highest proportion of active probesets (940 expressed probesets, 17% of the whole group). The lesser known PRIMA-41 group is the third most represented among gamma-retroviridae (581 probesets, 5% of the whole group). HML-8 (713 probesets) and HML-1 (178 probesets) are the groups with the highest proportion of expressed probesets (both 12%, of the whole corresponding group) among beta-retroviridae. The HML2 group, which is considered to be highly active, only showed 92 expressed probesets (9% of the whole group). Interestingly, although represented by a much smaller subgroup, centromeric HML2 elements seemed to be more expressed than the non-centromeric HML-2 ones. Finally, in spuma-retroviridae, HERV-L group provides the largest amount of active probesets (1197 probesets, 9% of the whole group). It should be noted that we did not observe significant enrichment or depletion of a specific repertoire, class or HERV group, according to healthy, inflammatory or immunocompromised-like experimental conditions (data not shown).

### Functional characterisation of the HERV transcriptome in PBMCs

**Fig. 2:**
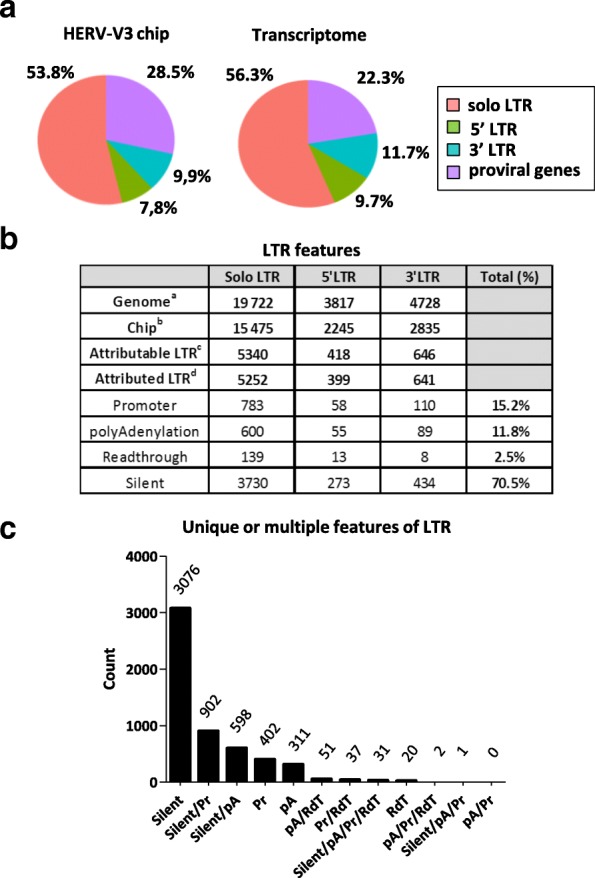
Genomic, transcriptomic and functional projections of the HERV prototype repertoire. **a** HERV structure distribution. The 42 HERV groups corresponding to the “HERV_prototypes” repertoire are voluntarily depicted as 100% of the HERV-V3 chip. Solo LTR, 5’ LTR, 3’ LTR and proviral genes account for 100% of the HERV chip in the proportion described in Additional file 2: Table S1b. The transcriptome pie-chart is obtained from results detailed in Additional file 2: Table S1c. **b** LTR features. The descriptive table summarises the assignment of features. ^a,b,c,d^ Loss of information from HERV database to understandable functions. ^a^ Summary of Additional file 2: Table S1a, ^b^ Summary of Additional file 2: Table S1b, ^c^ Enumeration of LTRs whose function is attributable, i.e. defined as LTRs combining U3 and U5 adjacent structures on the genome and existing probesets onto the chip allowing discrimination between U3 and U5 expression signals, ^d^ Enumeration of LTRs whose function is attributed using both a 2^4.5^ positive threshold and a fold change of 3 between U3 and U5 regions. More specifically, to retain sensitivity and robustness with regard to function assignation, we voluntarily selected a lower expression level cut-off of 2^4.5^ for positive signal attribution. As such, the LTR was referred to as promoter (Pr), polyadenylation signal (pA), readthrough (RdT) or Silent. All other remaining LTRs were classified as undetermined. **c** Specialisation of LTR features. Number of LTRs from the “HERV_prototypes” repertoire according to all the combinations of functions observed in each of the 45 PBMC samples. Silent LTR, Pr: LTR referred to as a promoter, pA: LTR referred to as a polyadenylation signal, RdT: read-through. The combinations obtained could either contain one (e.g. Pr), two (e.g. Silent/Pr), three (e/g. Silent/pA/Pr), or four functions (Silent/pA/Pr/RdT). 987 LTRs were excluded from the analysis as classified at least once as undetermined

Beyond the basic observation of transcriptional expression, it would be informative to know whether HERVs/MaLRs exhibit any transcription bias due to their structure. The HERV/MaLR transcriptomic activity observed collectively in stimulated and unstimulated PBMCs is depicted at the LTR/*gag*/*pol*/*env* region levels for each group/repertoire (Additional file 2: Table S1). Figure 2a provides a simplified comparative view of the “HERV_prototype” repertoire addressed by the microarray and its related transcriptome. We observed some differences between chip and transcriptome. Active LTRs represent 77.7% of the HERV transcriptome, whereas they represent 71.5% of the chip. This means that LTRs are more represented in active elements than internal HERV/MaLR regions. More specifically, solo LTRs are more abundant than proviral LTRs (5’LTRs and 3’LTRs). Nevertheless, it seems that proviral LTRs are over-represented in the transcriptome (21.4% versus 17.7%), whereas solo LTRs are not (56.3% versus 53.8%). Notably, internal proviral genes are under-represented in the transcriptome (22.3% versus 28.5%). Surprisingly, if we compare the “HERV_prototypes”, “HERV_Dfam” and “MaLR_Dfam” repertoire transcriptional activities, the HERV prototypes (14.3%) had higher numbers of expressed loci than Dfam elements (5.2% for HERVs and 4.4% for MaLRs). Attributable LTRs (solo, 5’ or 3’) are defined as LTRs bearing U3 and U5 regions. For these LTRs, we are able to attribute promoter or polyA functions. In the prototype repertoire, 6404 (31%) were attributable LTRs. According to cut-offs and fold change criteria (see material and methods), a function could be attributed to these LTRs in each of the 45 samples (Fig. 2b). Promoter (Pr) activity was assigned to 15.2% of LTRs and polyadenylation (pA) signal was observed in 11.8%. Most of the LTRs were silent (70.5%) and a minority (2.5%) were classified as readthrough (RdT).

Finally, we wanted to know whether a single LTR had the potential to change status (Fig. 2c). As expected, 56.6% of LTRs (3076 LTRs) were systematically silent in all samples. Interestingly, 27.6% of LTRs shifted from a silent status to a promoter (902 Silent/Pr LTRs) or polyadenylation (598 Silent/pA LTRs) function. Although poorly represented (2%), the same shift is observed for RdT LTRs. Some LTRs are exclusively RdT (20 LTRs) and some shifted from RdT status to a promoter (37 RdT/Pr LTRs) or polyadenylation (51 RdT/pA LTRs) function. A significant proportion of LTRs (13.1%) were in promoter (402 Pr LTRs) or polyA (311 pA LTRs) status in all samples (candidate loci validation, Additional file 8: Figure S6d). Almost no LTR (0.6%) shifted through at least three different features including both promoter and polyA functions. Consequently this observation confirms that the shift from promoter to polyA function is an extremely rare if significant event. The same analysis was performed using the complete HERV/MaLR dataset (Additional file 3: Figure S2). Although results should be considered with caution due to the imprecise annotation of HERV_Dfam and MaLR_Dfam LTRs (Additional file 2: Table S1), the overall trends were similar to those observed at the “HERV_prototypes” repertoire level.

The genomic environment encompassing the functional and silent LTRs is depicted in Additional file 4: Figure S3. As previously observed [29], the gene density ratio is almost 1.2 times higher for promoter LTRs than for silent LTRs. Meanwhile, the proportion of intragenic LTRs is biased towards the antisense representation, with about two-thirds of LTRs being antisense to the gene in which they are located, regardless of their functional category. About 80% of intragenic LTRs overlap with introns. Although no major bias in the genomic environment of intergenic LTRs (Additional file 4: Figure S3b) could be associated with their function, some trends are observed. A plateau is observed with constitutive promoter LTRs, reflecting a slightly lower occurrence of genes in the sense orientation up to 10 kb upstream of these LTRs. Symmetrically, sense gene occurrence apparently rises faster than for antisense genes in the downstream zone of silent LTRs compared to promoter LTRs.

### Gene and HERV/MaLR modulation following LPS stimulation

**Fig. 3:**
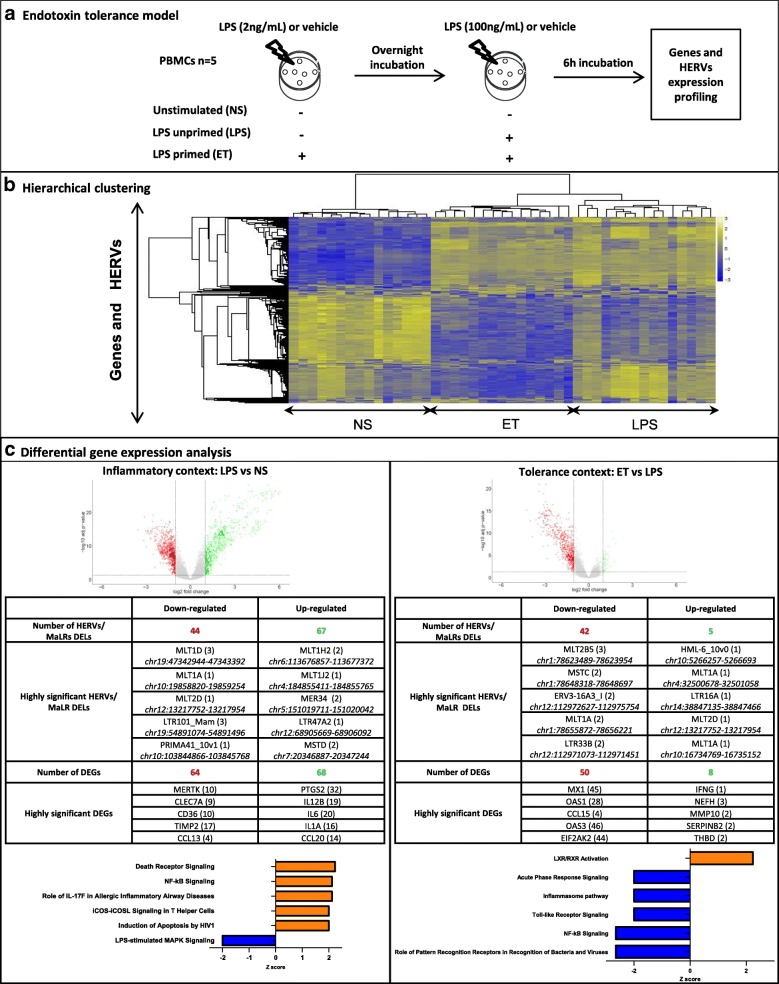
Genes and HERV/MaLR modulation following LPS stimulation. **a** Schematic representation of the dose-dependent LPS challenges known as the endotoxin tolerance model. Biological triplicates of PBMCs from 5 healthy volunteers were cultured and stimulated. Efficiency of stimulations was validated with TNF-α and IL10 quantitation by ELISA (Additional file 4: Figure S3) prior to HERV-V3 microarray experiments. **b** Heatmap from hierarchical clustering (correlation distance, complete method) of the 1% most variable probesets (all repertoires included), group samples according to their stimulation condition. Non-stimulated (NS), low-dose LPS primed PBMCs (ET), single high-dose lipopolysaccharide challenge (LPS); high-expression level (yellow), low-expression level (blue). **c** Differential gene and HERV/MaLR expression analysis. The first row shows volcano plots derived from the differential expression analysis; on the left for LPS vs NS (inflammatory context), and on the right for ET vs LPS conditions (immunocompromised/unresponsiveness context). The x-axis represents the log2 fold change values, and the y-axis the log10 adjusted *p*-values. Each point represents these values for a probeset. Coloured points show the significantly modulated probesets (adjusted p-value < 0.05, log2FC < − 1 (red) or log2FC > 1 (green)). The tables in the middle row present the number of statistically significantly differentially expressed elements, at locus level (DELs) for HERVs/MaLRs and differentially expressed genes (DEGs). Down-modulated loci are in red, up-modulated loci are in green. For HERV/MaLR elements, the name, number of differentially expressed probesets (between brackets) and chromosomal locations (in italic) are indicated (GRCh38 version of genome). For genes, the current gene symbol and the number of differentially expressed probesets (between brackets) are indicated. The last row represents canonical pathways identified using Ingenuity Pathways Analysis tool (https://analysis.ingenuity.com) and signals derived from HTA probesets contained on the HERV-V3 chip. Canonical pathways predicted to be significantly activated (orange) or inhibited (blue) between LPS vs NS and ET vs LPS conditions are depicted (z-scores ≥2 and z-scores ≤ − 2; *p*-value cut off of 0.05, Fisher’s exact test)

To gain insight into the modulation of genes and HERV/MaLR expression associated with PBMC stimulation conditions, we performed both a hierarchical clustering analysis and a supervised statistical analysis in pairwise conditions. For the record, PBMCs from 5 healthy volunteers were cultured in triplicate, (i) without any additional stimulation (NS for non-stimulated), (ii) with a high concentration of LPS to mimic inflammation, (iii) and primed with a low-dose LPS and latterly boosted with a high-dose LPS defining the so-called endotoxin tolerance model (ET) that mimics monocyte anergy (Fig. 3a). We checked our model by means of TNF-α pro-inflammatory and IL-10 anti-inflammatory cytokine quantification both in supernatants (protein level) and cellular extracts (mRNA level). The combined profiles of TNF-α as a “tolerisable gene” which exhibits a lower response, and as IL10, a “non tolerisable gene”, whose expression was increased or unaltered, validate the endotoxin tolerance model (Additional file 5: Figure S4). Figure 3b shows a heatmap from hierarchical clustering of the 1% most variant probesets (genes and HERVs/MaLRs) across samples. We observed 3 main expression profiles: (i) non tolerisable probesets with low expression for the NS condition and high expression for the ET and LPS conditions (top, Fig. 3b), (ii) tolerisable probesets with low expression for NS and ET, and high expression for the LPS conditions (bottom, Fig. 3b), and (iii) down-modulated probesets with high expression for the NS condition, and low expression for the LPS and ET conditions (middle, Fig. 3b). Hence, as stimulation conditions appeared to be strong drivers of PBMC transcriptome modulation, we performed a differential expression analysis. Differentially expressed genes are hereinafter referred to as DEG and differentially expressed HERVs/MaLRs loci referred to as DEL. Volcano plots are depicted at the probeset level for both genes and HERVs/MaLRs. They illustrated that similar amounts of elements appeared to be over- or under-expressed in an inflammatory context (LPS), whereas more gene and HERV/MaLR probesets were down-modulated under tolerance condition (ET) (Fig. 3c, top row).

Overall, differential expression analysis of LPS versus NS conditions identified 785 up-regulated probesets and 847 down-regulated probesets (corresponding to 243 distinct HERV/MaLRloci), while analysis of ET versus LPS conditions merely identified 38 up-regulated probesets and 677 down-regulated probesets (corresponding to 105 distinct HERV/MaLR loci) (adjusted *p*-value < 0.05, |log2FC| > 1) (Fig. 3c, middle, and Additional file 6: Table S2). Following LPS stimulation, out of the 111 differentially expressed HERV/MaLR elements, 38 belong to HERV_Dfam, 51 to MaLR_Dfam, and 22 to HERV_prototypes. Although DEL analysis does not indicate any group or chromosomal enrichment, some characteristic points could be observed. ERV9, ERV-E4.1, HERV-FRD, HERV-H, HERV-I, HML8, PABL-A and PRIMA4 groups were exclusively modulated under inflammatory condition (LPS vs NS). HERV-Fb, HERV-L, HERV-T and PRIMA41 groups were both modulated for LPS vs NS and ET vs LPS (see below). Among the DELs, 64% consist of solo LTRs and 36% of complete or partial proviruses. After LPS stimulation, PTGS2, IL1B, IL12B, IL6, IL1A and TNF-α pro-inflammatory cytokine as well as CCL20 and PTX3, were the most up-modulated genes. MERTK, CLEC7A, CD36, TIMP2 and CCL13 were the most down-modulated genes. Moreover, we observed, with a pathway analysis, an enrichment of the Death Receptor Signalling and NF-ҡB Signalling pathways (Fig. 3c, last row). Notably, these results highlighted significant inactivation of “LPS-stimulated MAPK signalling”. This may be due to negative feedback resulting from the high production of TNF-α following LPS stimulation.

In a tolerance context, among the 47 differentially-expressed HERV/MaLR elements, 20 belonged to HERV_Dfam, 20 to MaLR_Dfam and 7 to HERV_prototypes. As observed for LPS vs NS, no group is enriched or depleted among the most DELs. Notably, 11 elements out of 47 (23.4%) are located on chromosome 12. The HERV-HS49C23, HERV-F and HML6 elements were exclusively modulated in the ET vs LPS condition. Some groups had differentially expressed loci in LPS vs NS and other differentially expressed loci in ET vs LPS, such as HERV-Fb, HERV-L and HERV-T groups. Among the 47 DELs, 86% consist of solo LTRs and 14% of complete or partial proviruses. In a tolerance context, IFNγ, NEFH, MMP10, SERPINB2 and THBD were the most up-modulated genes. MX1, OAS1, CCL15, OAS3, EIF2AK2 and TNF-α were the most down-modulated genes. Consistently, there were 3 inhibited pathways: “role of pattern recognition receptors in recognition of bacteria and viruses”, “NF-κB signalling” and “TLR signalling”(Fig. 3c, last row). Conversely, “LXR/RXR signalling” was highly activated, putatively reflecting LXR-induced inactivation of the NF-ҡB signalling pathway leading to the anti-inflammatory macrophage phenotype in atherosclerosis (Fig. 3c, last row; [54]).

Differential expression analysis allowed us to identify modulated HERVs/MaLRs and genes involved in inflammatory and tolerance contexts. Before considering functional linking of HERVs/MaLRs and genes, we validated the results obtained with microarrays through the use of the RT-qPCR reference method.

### RT-qPCR confirmation of condition-related expression of HERV/MaLR elements

To confirm modulation of expression observed with the HERV-V3 microarray, we selected the 44 HERV/MaLR probesets for RT-qPCR validation,. Of these, 31 were the most differentially expressed probesets in Dfam repertoires and 13 the most differentially expressed probesets among the prototype repertoire. HERV/MaLR locus-specific RT-qPCR systems were meticulously designed and validated to secure locus specificity (see Additional file 7: Figure S5), leading to 32 primer pairs out of 44 of selected candidates. We confirmed HERV/MaLR modulation on the samples used for microarray analysis, and then on an independent cohort of 6 healthy volunteers. PCR products could be obtained on 23 out of 32 primer pairs (depicted in Additional file 7: Figure S5). Overall, 87% of the detectable elements had concordant profiles with HERV-V3 microarray data. Twenty loci exhibited similar expression profiles in microarray and RT-qPCR experiments and 3 exhibited conflicting profiles (Additional file 8: Figure S6).

**Fig. 4:**
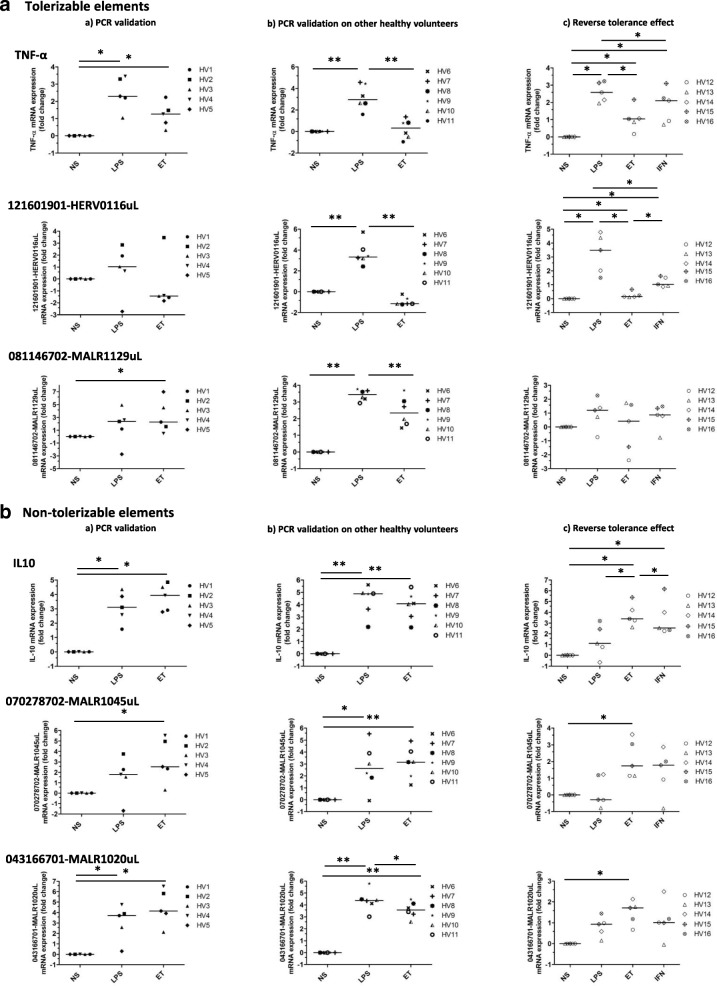
RT-qPCR validation of differentially expressed candidate loci following various LPS dose challenges. This figure illustrates the RT-qPCR expression of **a** tolerisable genes (TNF-α) and HERV/MaLR elements (121601901-HERV0116uL and 081146702-MALR1129uL) exhibiting a TNF-α–like pattern of expression and **b** non-tolerisable genes (IL10) and HERV/MaLR elements (070278702-MALR1045uL and 043166701-MALR1020uL) exhibiting an IL10–like pattern of expression. Expression was measured using mRNA derived from the stimulated and unstimulated PBMCs of the healthy volunteers used in the discovery microarray experiment (first column, (Aa and Ba)), mRNA derived from the stimulated and unstimulated PBMCs of 6 additional healthy volunteers managed in the same way (second column, (Ab and Bb)), and finally mRNA derived from the stimulated and unstimulated PBMCs of 5 additional healthy volunteers (third column, (Ac and Bc)). This last column includes additional IFN-γ dependant reversibility of tolerance, consisting of a 2 ng/mL LPS priming step overnight, followed by a 100 ng/mL IFN-γ stimulation step overnight, and finally the 100 ng/mL LPS stimulation step for 6 hours. All PCR reactions were performed in duplicate for each condition. Expression of the housekeeping genes PPIB and RPLP0 was monitored for normalisation. The fold change (FC) was determined using the 2^-ΔΔCt^ method. The final value of the unstimulated condition was arbitrarily set to one and other values scaled-up in order to provide a final relative differential expression (data were represented by a median and using the log2 scale). Statistically significant differences between two conditions are marked (wilkoxon signed rank test. **: *p*-value < 0.05 and * *p*-value < 0.1)

Notably, a majority of HERV/MaLR elements exhibited similar patterns as “tolerisable” or “non tolerisable” genes, i.e. divergently modulated following LPS and ET treatments. Figure 4a illustrates the comparable “tolerisable” behaviour of TNF-α, 121601901-HERV0116uL, and 08114670-MALR1129uL HERV loci. Figure 4b depicts the similar “non tolerisable” behaviour of IL10, 070278702-MALR1045uL, and 043166701-MALR1020uL MaLR loci. These two phenotypes were observed regardless of their distance from genes. Nevertheless, 121601901-HERV0116uL, 070278702-MALR1045uL, and 043166701-MALR1020uL are located within OAS3, ITGB8 and MIR3945HG genes, respectively. Conversely, 08114670-MALR1129uL is at a distance of more than 100 kb from the closest gene. Interestingly, as known for TNF-α [51], the tolerisable HERV phenotype was reversed by IFN-γ (Fig. 4a and b, column c). Hence, as HERVs/MaLRs and genes seemed to share similar control of expression following stimulation, we attempted to integrate HERVs/MaLRs in gene pathways based on their common transcriptional behaviour.

### An integrative view of HERVs/MaLRs and genes in immunity pathways

Differential expression analysis identified genes and HERV/MaLR loci for which expression was modulated in inflammatory or tolerance contexts. Some HERVs/MaLRs and genes had strongly correlated expression profiles. We sought to identify which HERV elements may be co-expressed with genes which were integrated in regulatory networks. First, using Ingenuity Pathway Analysis (IPA), we identified 13 activated or repressed canonical pathways in inflammatory or tolerance contexts. In 11 out of the 13 pathways, 26 genes belonged exclusively to one pathway. We then identified 72 probesets corresponding to 62 HERV/MaLR loci strongly correlated with the 26 genes (correlation ≥0.8) (Additional file 9: Figure S7a-c, Additional file 10: Table S3). Among those 62 HERV and MaLR loci, 7, 26 and 29 belonged to “HERV_prototypes”, HERV_Dfam and MaLR_Dfam repertoires, respectively. This allowed us to build a global network integrating HERV and MaLR loci within gene pathways (see Additional file 9: Figure S7d). Twenty-six out of the 62 HERV/MaLR elements belonged exclusively to one pathway, and 36 HERV/MaLR loci are associated with 2 to 7 pathways. Eleven HERV or MaLR loci were identified in the vicinity of genes (≤ 40 kb), and they were integrated into 1 to 4 pathways. Conversely, although spread on various chromosomes, a bundle of 8 HERV/MaLR elements contributed to both the LXR/RXR activation pathway activated under ET condition and the NF-κB signalling pathway activated during inflammation, via PTGS2 and FLT1 genes (see Additional file 9: Figure S7e).

**Fig. 5:**
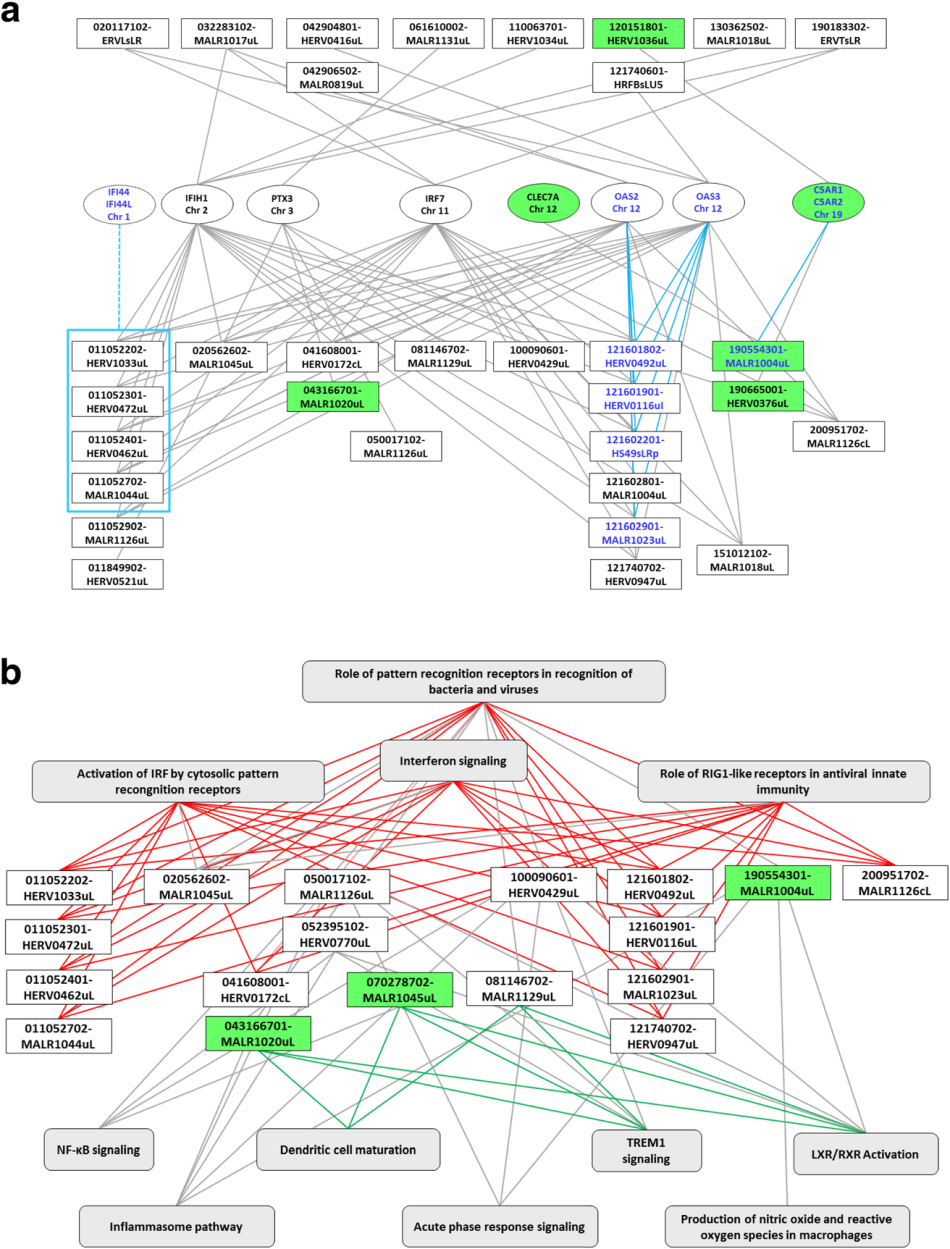
Expression profile-based integration of HERVs/MaLRs in immunity genes related pathways. **a** The gene-centred network illustrates HERV/MaLR integration in the “role of pattern recognition receptors in recognition of bacteria and viruses” pathway (PRR). Seven genes specific for this pathway are depicted (oval frames) together with their parental chromosome. HERV/MaLR elements associated with these unique genes were identified using both Ingenuity Pathway Analysis and co-expression between HERVs/MaLRs and genes. Tolerisable and non-tolerisable elements are represented by empty or green boxes, respectively. Ten HERV/MaLR elements belong exclusively to the PRR pathway (top rectangular frames), while 22 HERVs/MaLRs loci are shared by several pathways (bottom rectangular frames). Five HERV/MaLR elements are located at a distance of less than 40 kb from the OAS3 and OAS2 or C5AR1/C5AR2 genes (dark blue font and turquoise connectors, C5AR2 absent from the microarray). Four HERV/MaLR elements are located in a 40 kb window (large turquoise rectangle) flanked by IFI44 and IFI44L genes (connected by a dotted line as absent from the microarray). **b** HERVs/MALRs integration in several pathways. The HERV/MaLR-centred network illustrates the integration of HERVs and MaLRs elements within 11 networks (grey boxes). Tolerisable and non-tolerisable elements are represented by empty or green boxes, respectively. Ten out of 15 tolerisable HERV/MaLR elements are exclusively linked to the 4 pathways at the top (red connectors), “activation of IRF by cytosolic pattern recognition receptors”, “role of pattern recognition receptors in recognition of bacteria and viruses”, “interferon signalling” and “role of RIG1-like receptors in antiviral innate immunity”. Three elements are exclusively associated with 3 pathways (2 out of the 3 non-tolerisable elements and 1 tolerisable element), “dendritic cell maturation”, “TREM1 signalling”, and “LXR/RXR activation” (green connectors)

A gene centred view of the “Role of pattern recognition receptors in recognition of bacteria and viruses” (PRR) pathway is depicted in Fig. 5a. It includes 32 HERV/MaLR loci and 7 genes exclusive to the pathway. Ten out of the 32 HERV/MaLR loci belong exclusively to this pathway. Most of the genes are co-expressed with several retroviral elements, 18 for IFIH1, 18 for IRF7, 17 for OAS3, 15 for OAS2, 7 for PTX3 and 3 for C5AR1, widespread on distinct chromosomes. Interestingly, 4 tolerisable HERV/MaLR loci are present on chromosome 12 within a 39 kb region which overlaps with OAS2 and OAS3 tolerisable genes. Moreover, 4 HERV/MaLR loci are on chromosome 1 in a 32 kb region overlapping with IFI44L and IFI44 genes. RT-PCR amplification of IFI44L, IFI44 genes and 011052301-HERV0472uL HERV loci showed a marked decrease for the ET condition (data not shown). Notably, most of these HERV/MaLR elements expressed belonged to the 3’UTR of several gene transcripts: 121601802-HERV0492uL and 121601901-HERV0116uL for OAS3–201, 121602201-HS49sLRp for OAS2–203, 121602901-MALR1023uL for OAS2–202, and 011052301-HERV0472uL and 011052401-HERV0462uL for IFI44L-201.

Alternatively, an HERV/MaLR-centred view is proposed in Fig. 5b and highlights 18 loci which belong to at least 4 different networks. Eleven out of 15 HERV/MaLR tolerisable loci were shared by 4 pathways (PRR, RIG-1, Interferon and IRF pathways). These 11 elements were co-localised with or close to conventional genes (≤ 10 kb). Non-tolerisable elements 070278702-MaLR1045uL and 043166701-MaLR1020uL and tolerisable element 081146702-MALR1129uL are at the crossroad of several pathways, namely “LXR/RXR activation”, “TREM1 signalling”, and “Dendritic cell maturation” Altogether, this interplay between HERVs/MaLRs, genes and pathways suggested complex and heterogeneous transcriptional regulation mechanisms.

## Discussion

### HERV transcriptome in PBMCs

We used the HERV-V3 chip to provide a first overview of the HERV transcriptome in PBMCs. Healthy, inflammatory and immunocompromised/monocyte anergy states were simulated using an endotoxin tolerance model [50]. We observed that about 5.6% of LTR retrotransposons were transcriptionally active in PBMCs, reaching 9.4% in the well-annotated “HERV_prototypes” repertoire. Previous works estimated the extent of the HERV transcriptome, compared to the HERV genome, to be between 7 and 30% [29, 55–57]. These differences could be related to (i) disease conditions [29, 55, 57], (ii) tissue specificity [29, 55], and (iii) technology [29, 55–57]. Although various intensity thresholds (background/expressed) may also account for such differences, we confirmed in our study that a significant amount of HERV and MaLR sequences thought to be silent are actually expressed. HERV or MaLR regions might be either embarked by conventional genes, or self-induced. Indeed, an LTR could possess promoter activity or harbour polyadenylation signals used for proviral gene expression or adjacent non-retroviral genomic sequences. The vast majority of LTRs were silent (70.5%), and the minority were transcribed via readthrough mechanism (2.5%), regardless of the condition. The large amount of silent LTRs is in line with the accumulation of inactivating mutations [58] as well as silencing by epigenetic mechanisms [59, 60]. A relatively balanced state was observed between putative promoter (15.2%) and polyadenylation (11.8%) functions. The same overall trends were previously observed in cancer tissues [29], although the amount of silent LTRs was higher in our study (70.5% versus 47%), probably due to the largely increased number of targeted groups. Altogether, integrating both more precise data derived from the “HERV_prototype” repertoire and results from the complete HERV/MaLR dataset, about one tenth of LTRs exhibit a constitutive promoter or polyA function while roughly one quarter of LTRs may shift between silent and promoter or polyA functions. Only a few thousandths of LTRs can shift between promoter and polyA, as initially described in cancer [29], strengthening what we called “operational determinism”, i.e. an LTR is predetermined to act as a promoter or a polyA site. Although all “prototype” groups are active in PBMCs, we observed a higher proportion of gamma-retroviruses, including notably the super spreader HERV-H group and the HERV-W group containing Syncytin 1. This is consistent with group-based PCR approaches in PBMCs [36], and in MDM cell lines [41], as well as with the detection/modulation of expression of MSRV/HERV-W and HERV-H loci in PBMCs of healthy donors and multiple sclerosis patients [15, 45, 61, 62]. Concerning beta-retroviruses, HERV-K/HML elements were expressed in PBMCs as previously observed in healthy subjects [36, 41], in prostate cancer [37], and Henoch-Schönlein purpura [63] patients.Notably, centromeric HML2 elements seemed to be relatively more represented than the other HML-2 elements, as observed in the blood of HIV-infected patients [64]. Nevertheless, this exhaustive HERV-dedicated microarray allowed us to detect expression of poorly characterised groups such as PRIMA41 and MER52A which merit further investigations.

### Modulation of HERVs and genes after inflammation and tolerance induction

After looking at the HERV transcriptome landscape in PBMCs, we analysed whether individual HERVs/MaLRs could be finely regulated upon stimulation. Hierarchical clustering aggregated samples according to their stimulatory status and DE analysis showed that many HERVs/MaLRs and genes were modulated between conditions. We observed a similar amount of up-regulated and down-regulated elements in the LPS condition compared to NS, as previously shown using group-based RT-PCR systems [36, 41]. Such dichotomic activity of HERVs/MaLRs was found in the highly inflammatory context of burn patients [65, 66]. In mice, LPS stress induced primary lymphoid cell-specific production of MLV-ERV virions [67]. More generally, microbes modulated ERV transcription in mice [42]. In a tolerance context, HERVs/MaLRs and genes tended to be down-regulated. Altogether, these observations suggest various levels of HERV/MaLR control, including a fine-tuned control similar to conventional genes.

The NF-ҡB signalling pathway was both up-regulated following LPS stimulation and down-regulated following tolerance induction (LPSvsNS, Z score: 2.121; ETvsLPS, Z score: − 2.646). This is highlighted by the pro-inflammatory cytokines, IL12B, IL6, IL1A, TNF-α modulation, as well as IL10 anti-inflammatory variation, as previously described in such contexts [50, 68–70]. As observed for TNF-α and IL10 genes, HERV/MaLR elements exhibit a dichotomy of tolerisable versus non tolerisable phenotypes, although some elements were found to be down-regulated in both conditions. As illustrated by 121601901-HERV0116uL and 081146702-MALR1129uL loci, HERV/MaLR tolerisation was surprisingly reversible upon IFN-γ addition, as previously described for TNF-α [51, 52]. Again, this demonstrates that HERVs/MaLRs and genes share similar regulation control following stimulation inducing inflammation or anergy in PBMCs. These results confirm the existence of tight HERV expression regulation control, as previously suggested by the tropism-related behaviour of HERV elements in solid tissues and in particular in reproductive tissues [3] and cancer [29].

### HERV integration within gene immunity pathways

The comparable responses of HERVs/MaLRs and genes to high-dose, low-dose LPS and IFN-γ-dependent reversibility of tolerance may be due to either autonomous HERV expression potentially driving gene expression, or gene expression potentially embedding HERV expression. Pathway and co-expression analyses allowed us to include 62 HERV/MaLR loci and 26 genes which are exclusive to 1 pathway into regulatory networks. Eleven canonical pathways were activated or repressed in inflammatory or tolerance contexts. Among the integrated HERV/MaLR elements, ten loci mapped to the 3’UTR of OAS2, OAS3, IFI44L, IFI44, C5AR1 and C5AR2 genes. These retroviral elements may contribute to the post-transcriptional control of these transcripts, including polyA signalling, nucleocytoplasmic transport, translation efficiency, localisation and stabilisation of mRNA [71]. The complex interplay of HERVs and genes can be illustrated by the IFI44L to IFI44 gene region on chromosome 1. The 2 genes and the 4 HERV/MaLR loci located between them are similarly down-regulated under ET condition. Such similar transcriptional expression of IFI44L and IFI44 was previously described in purified CD14 monocytes of patients with Sjogren’s syndrome [72, 73]. Interestingly, dissociated IFI44L versus IFI44 expression was observed in high versus low IFN-γ producers following *Leishmania braziliensis* stimulation in PBMCs [74]. In addition, among the 4 HERV/MaLR elements, the 011052702-MALR1044uL locus corresponded to an annotated CTCF binding region which defines the boundary between active and heterochromatic DNA. The 011052702-MALR1044uL locus may therefore regulates IFI44L and IFI44 expressions in some situations.

The co-expression of genes with a large number of HERV/MaLR elements scattered among different chromosomes, e.g. 18 HERV/MaLR loci located on 10 distinct chromosomes linked with IFIH1, 6 HERVs/MaLRs located on 6 distinct chromosomes linked with PTX3, suggested that they are part of gene networks regulated by shared transcription factors, as previously proposed [33]. However, we did not observe any tissue-specific transcriptional factor binding sites (TFBS), but an enrichment of the AP-1 binding site with promoter LTR was observed as compared to silent LTR (*p*-value: 1.14 10^− 4^, data not shown). The similar modulation of MALR1126 LTR promoters belonging to LXR/RXR and NF-κB networks and located on chromosome 5 (050017102-MALR1126uL) and 10 (100175702-MALR1126uL) may reflect such shared regulation. The integration of LTRs within several pathways is a first suggestion that HERVs/MaLRs and genes are similarly regulated. The HERVs/MaLRs integrated in PRR, RIG1, IFN and IRF pathways and which are mapped into gene transcripts, are mostly tolerisable and carry H3K36me3, a mark of actively transcribed regions in normal haematopoietic cells. Most HERV/MaLR elements, either tolerisable or non-tolerisable and integrated into LXR/RXR, TREM1 and dendritic cell maturation or LXR/RXR and NF-ҡB pathways, are at a distance of more than 25 kb from the closest gene and carry mainly H3K27me3, and occasionally H3K9me3, mark of repressed regions in normal haematopoietic cells. Notably, the IFI44-associated 011052702-MALR1044uL and SLC30A4-associated 150312301-HERV0498uL loci appeared to be decorated with H3K9me3 and H3K27me3 repressive histone marks, respectively. As a general trend, LTRs were screened for particular histone modification signals by overlap with Encode peaks for H3K27ac, H3K27me3, H3K36me3, H3K4me1, H3K4me3 and H3K9me3 in different immune cells; we found enrichment for the H3K9me3 mark for CD14+ monocytes. The LPS stimulations probably modified the local chromatin configuration leading to global modulation of expression, of the gene and HERV/MaLR [75]. Intriguingly, it appears that CD14-positive monocytes are at least twice more enriched in H3K9me3 histone mark than CD4- and CD8-positive T cells, B cells and natural killer cells (data not shown). Taken together, the variability of histone marks, the tolerisable/non-tolerisable HERV/MaLR phenotypes and vicinity of genes suggest a common control of expression between HERV/MaLR and genes. In addition to a contribution to cis-regulation, HERV/MaLR presence at the crossroads of different regulatory networks and conservation in the human population (data not shown), may suggest a role in trans-regulation linking enhancer and promoter regions [33, 76]. A better understanding of the causal relationship between HERV/MaLR, genes and regulatory pathways would merit further investigations.

## Conclusion

This microarray-based approach revealed the expression of about 47,466 distinct HERV loci and identified 951 putative promoter LTRs and 744 putative polyA LTRs in PBMCs. HERV/MaLR expression was shown to be tightly modulated following several stimuli including high-dose and low-dose LPS as well as IFN-γ. This allowed us to propose an integrative view of HERVs/MaLRs and genes in global functional pathways. Further systematic analyses will be required to gain insight on the modulation of expression of HERV/MaLR loci in different haematopoietic cell types, including monocytes, B and T cells, as well as neutrophils and NK cells. This may help decipher the multiple levels of HERV functions in haematopoietic cells, as locally illustrated by the surface or intracellular envelope on monocytes [45] or glial cells [77], or by non-coding elements involved in the control of cell differentiation [78]. From an in vivo point of view, this approach paves the way for systematic deciphering of modulated retroviral elements associated with autoimmune diseases such as systemic lupus erythematosus, inflammatory diseases such as type 1 diabetes [79] inherited autoimmune and auto-inflammatory disorders such as type 1 interferonopathies (reviewed in [80, 81]), and virus- or drug-induced immunocompromised states [82], as well as resulting from a compensatory response to hyperinflammation such as in sepsis [83]. Notably, it would be of interest to investigate whether the altered histone methylation recently observed for genes in LPS-induced tolerance and in septic patients [75, 84] may affect HERV expression and contribute to sepsis.

## Methods

### Biological samples and quality control

Citrated pouches or heparinised tubes blood were obtained from EFS (Etablissement Français du Sang) and used immediately. According to EFS standardised procedures for blood donation and to provisions of the articles R.1243–49 and following ones of the French Public Health Code, a written non-opposition to the use of his donation for research purposes was obtained from healthy volunteers. The blood donors’ personal data were anonymised before blood transfer to our research lab. We obtained the favourable notice of the Local Ethical Committee (Comité de Protection des Personnes Sud-Est II, Bâtiment Pinel, 59 Boulevard Pinel, 69,500 Bron) and the acceptance of the Ministère de la Recherche (declaration DC-2008-64) for handling and conservation of these samples. Peripheral blood mononuclear cells (PBMCs) were isolated with Unisep tube density gradient centrifugation (Eurobio) and washed with sterile PBS (phosphate buffered saline) (Eurobio). The PBMCs were adjusted to 2 × 10^6^ cell/mL and cultured in X-Vivo 20 Medium (Lonza) at 37 °C and 5% CO_2_. All the experiments were perfomed in triplicate. Lipopolysaccharide was purchased from Sigma-Aldrich and was a mix of *Escherichia coli* O111:B4, O55:B5 and O127:B8 (Sigma). In this ex vivo endotoxin tolerance model, the PBMCs were first cultured for 15 h without (control group NS and LPS cells), or with 2 ng/ml LPS (ET cells). After washing steps, the PBMCs were incubated a second time for 6 h without (control group NS), or with 100 ng/ml LPS (LPS and ET cells) (Fig. 3a). In this model, when specified in the text, the effects of recombinant human IFN-γ to reverse tolerance effects were studied. Another incubation phase was performed for 24 h with 100 ng/mL of human IFN-γ1b (Miltenyi Biotec) or vehicle, between the two LPS incubations. At the end of the experiments, the supernatants were retrieved and stored at − 80 °C. Pro-inflammatory cytokine TNF-α and anti-inflammatory cytokine IL10 concentrations in the PBMC culture supernatants were detected using commercially-available ELISA kits from R&D System, in accordance with the supplier’s recommendations. The cells were harvested, lysed in RLT buffer supplemented with β mercaptoethanol and stored at − 80 °C until further processing. The total RNA was extracted from PBMCs using RNeasy Mini kit (Qiagen) according to the manufacturer’s instructions. For each RNA extraction, the residual genomic DNA was digested using the gDNA Eliminator spin column (Qiagen), and directly on RNeasy spin column using RNase-Free DNase Set (Qiagen). RNA quantity and quality were determined using Nanodrop (Thermo Scientific) Bioanalyser 2100 (Agilent) according to the manufacturer’s instructions [51, 52].

### Custom Affymetrix HERV-V3 GeneChip microarray

HERV-V3 targets 353,994 loci-elements, represented by 4,410,200 probes. The custom HERV GeneChip can discriminate between distinct HERV elements composed of a set of highly informative probesets (located in U3, R, U5 subdomains of solo, 5’ and 3’ individual LTRs and *gag*/*pol*/ *env* regions), hereinafter referred to as ‘HERV prototypes repertoire’, and a set of probesets with lower-quality annotations (located in the first third and last third of the complete LTR, and every 2.5 kb in the region in between LTRs), hereinafter referred to as ‘HERV/MaLR_Dfam repertoire’. The custom HERV GeneChip also contains probesets targeting LINE1, lncRNA, viruses, and the gene repertoire. The descriptions of the HERVgDB4 database and of the final contents of the HERV-V3 microarray are provided in Additional file 2: Table S1 [30].

### RNA amplification, labelling and hybridisation

The cDNA synthesis and amplification steps were performed using 16 ng of RNA with the Ovation Pico WTA System V2 kit (Nugen) according to the manufacturer’s instructions. Five micrograms of amplified purified DNA were fragmented into 50–200 bp fragments and were 3-labeled using the Encore Biotin Module kit (Nugen) according to the manufacturer’s instructions. The HERV-V3 microarrays were hybridised at 50 °C for 18 h in an oven with constant stirring (60 rpm). Washing and staining were carried out according to the protocol provided by the manufacturer, using the GeneChip fluidics station 450 (Affymetrix). The arrays were finally scanned using the GeneChip scanner 3000 7G (Affymetrix) fluorometric scanner. Images (DAT files) were converted to CEL files using GCOS software (Affymetrix) [30]. The experimental data generated have been filed with the National Center for Biotechnology Information (NCBI) and are available on the GEO DataSets site under access number GSE108239.

### Bioinformatics analysis

Microarray analysis pre-processing was detailed in supplementary methods (Additional file 11: Supplementary Methods). We chose a specific threshold to define LTR functions for the HERV_Dfam repertoire. We used the dichotomy of probeset signal targeting. More specifically, to retain sensitivity and robustness with regard tofunction assignation, we voluntarily and arbitrarily selected a relatively low expression level cut-off of 2^4.5^ for positive signal attribution coupled with a significant fold change between U3 and U5 signals. Therefore, an LTR was referred to as ‘promoter’ (Pr) in cases where the signal of the U5-associated probeset was (i) over the threshold, and (ii) at least 3 times higher than its U3 counterpart, and as ‘polyadenylation signal’ (pA) if the intensity of its U3-associated probeset was (i) over the threshold and (ii) at least 3 times higher than its U5 counterpart. An LTR was assigned as ‘readthrough’ (RdT) if both U3 and U5 signals (i) were over the threshold, and (ii) without significant fold change between its. Finally, an LTR was assigned as silent if U3 and U5 were both under the threshold; all other remaining LTRs were classified as undetermined (112 LTRs). To visualise HERV and gene co-expression, hierarchical clustering based on correlation distance with the average method was performed on the 1% most variable probesets. Subsequently, comparisons between i) unstimulated PBMCs (NS) and PBMCs stimulated once with LPS (LPS), and ii) tolerant PBMCs re-stimulated with LPS (ET) and LPS were carried out. For all probesets, for differential expression analysis, moderated t-tests were performed (Limma, v3.22.7 [85], and *p*-values adjusted for multiple testing using the Benjamini-Hochberg procedure [86]. A probeset was considered to be statistically significantly differentially expressed when the absolute log2 Fold Change (|log2FC|) was over 1 and the adjusted-p-value under 0.05. Graphs were generated using ggplot2 (v2.2.0) or pheatmap (v1.0.8). Finally, the Ingenuity Pathways Analysis tool (IPA, Ingenuity® Systems, https://analysis.ingenuity.com) was used to assess upstream regulators, canonical pathways, disease, and functions. Details of this analysis were presented in supplementary methods (Additional file 11: Supplementary Methods). HERVs with a correlation coefficient of over 0.8 with an identified gene were selected for integrative pathway view analysis.

## Additional files

Additional file 1: **Figure S1.** Definition of the positive intensity threshold. (PPT 279 kb)

Additional file 2: **Table S1**. Detection of the HERV transcriptome in PBMCs. (XLSX 145 kb)

Additional file 3: **Figure S2.** Specialisation of LTR features on the whole dataset. (PPT 212 kb)

Additional file 4: **Figure S3.** Genomic environment of functional and silent LTRs. (DOC 507 kb)

Additional file 5: **Figure S4.** TNF-α and IL-10 protein assay and mRNA quantitation in PBMCs following LPS stimulations. (PPT 172 kb)

Additional file 6: **Table S2**. Differential expression of HERV-V3 chip repertoires induced by stimulation state changes in endotoxin tolerance model. (XLSX 41775 kb)

Additional file 7: **Figure S5.** Selection, design and quality criteria for the design of locus specific qPCR systems, illustrated with the 121601901-HERV0116uL locus, and PCR systems obtained. (PPT 766 kb)

Additional file 8: **Figure S6.** RT-qPCR validation of 23 microarray-based identified HERV/MaLR elements differentially expressed following LPS stimulations. (PPT 1309 kb)

Additional file 9: **Figure S7.** Strategy used to integrate 62 HERVs/MaLRs and 26 genes in 11 canonical immune pathways, global landscape resulting from this analysis, and identification of PTGS2 and FLT1 associated HERVs/MaLRs at the crossroads of “LXR/RXR activation” and “NF-kB signalling” pathways. (PPT 1323 kb)

Additional file 10: **Table S3.** HERVs or MaLRs with the same expression profiles as genes within pathways. (XLS 53 kb)

Additional file 11: Supplementary Methods. (DOC 55 kb)

## Abbreviations

DEG: Differentially expressed gene
DEL: Differentially expressed locus
ET: Endotoxin tolerance
HERV: Human endogenous retrovirus
IFN: Interferon
IPA: Ingenuity pathway analysis
LPS: Lipopolysaccharide
LTR: Long terminal repeat
MaLR: Mammalian apparent LTR-retrotransposons
MSRV: Multiple sclerosis associated retrovirus
NS: Non stimulated
pA: Polyadenylation
PBMC: Peripheral blood mononuclear cell
Pr: Promoter
