# Supplementary material for: LTR-retrotransposon transcriptome modulation in response to endotoxin-induced stress in PBMCs
Source: BMC Genomics. 2018 Jul 5;19:522. doi: 10.1186/s12864-018-4901-9 (PMC6034278; doi:10.1186/s12864-018-4901-9)
Supplement: Supplementary file 1 — Figure S1. Definition of the positive intensity threshold. (PPT 279 kb) [file 12864_2018_4901_MOESM1_ESM.ppt]

## Slide 1
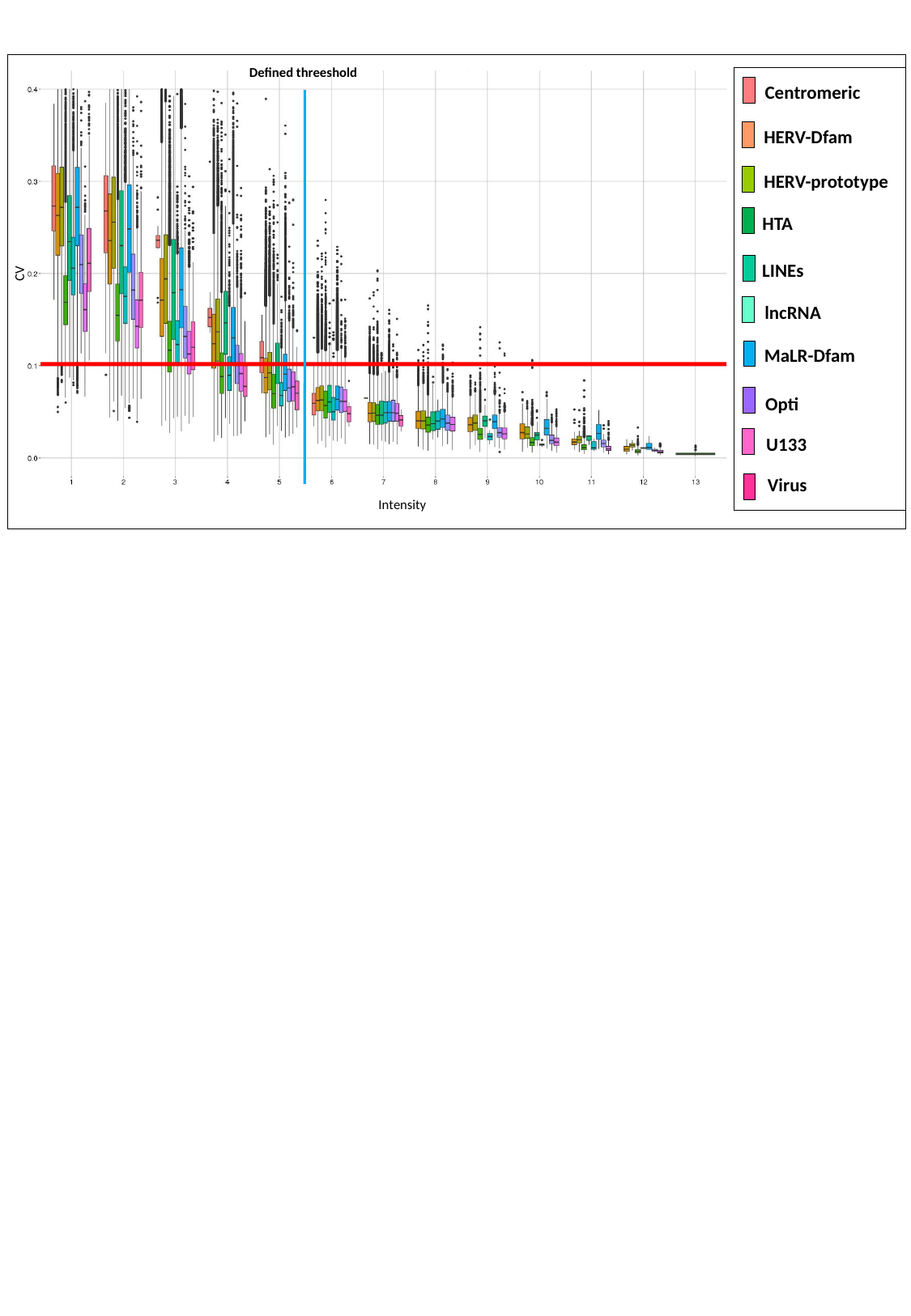

Defined threeshold
Centromeric
HERV-Dfam
HERV-prototype
HTA
LINEs
CV
lncRNA
MaLR-Dfam
Opti
U133
Virus
Intensity
