# Supplementary figures and images for: LTR-retrotransposon transcriptome modulation in response to endotoxin-induced stress in PBMCs
Source: BMC Genomics. 2018 Jul 5;19:522. doi: 10.1186/s12864-018-4901-9 (PMC6034278; doi:10.1186/s12864-018-4901-9)

## Slide 1
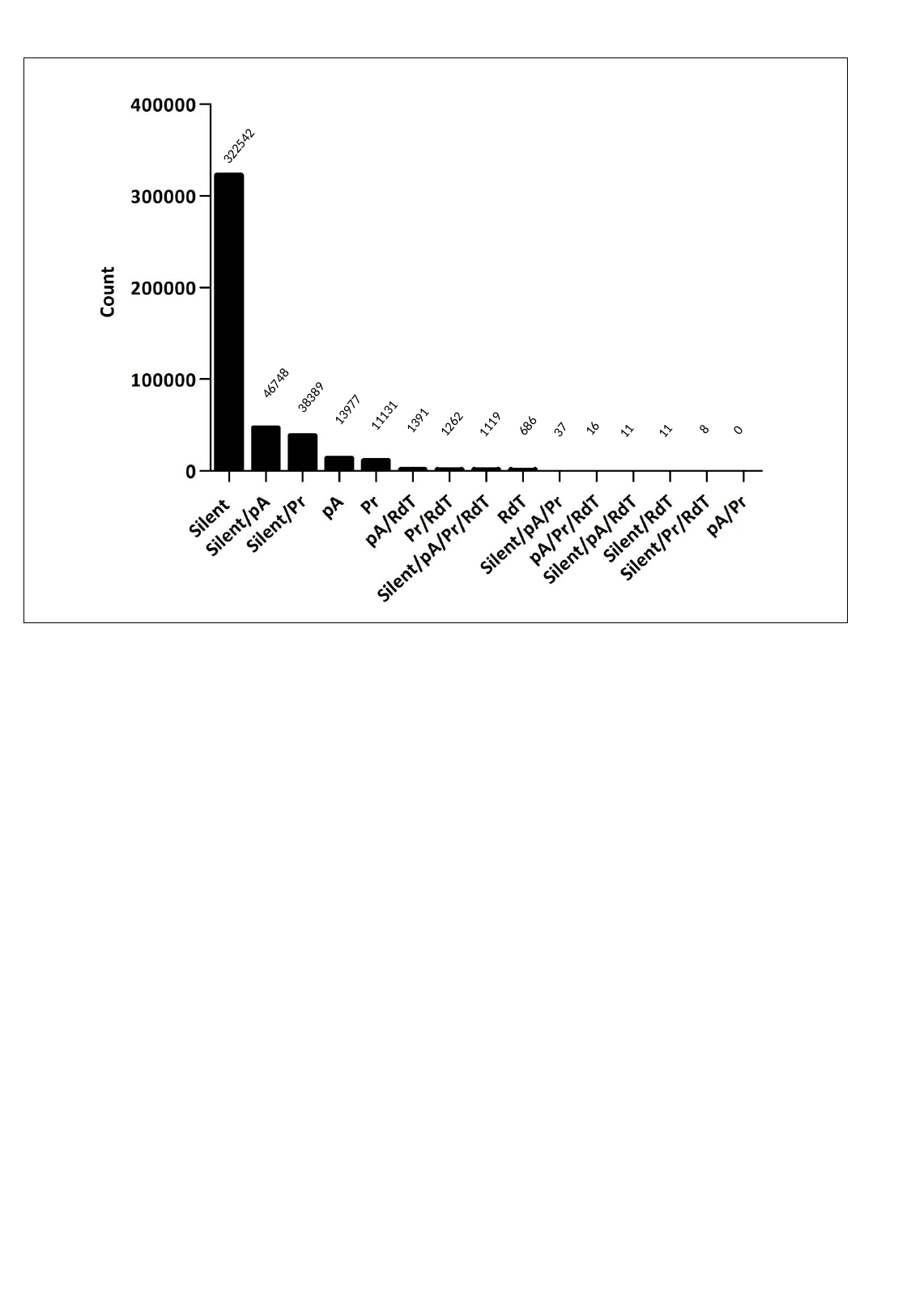

322542
46748
38389
13977
11131
1391
8
0
686
16
1262
1119
37
11
11

Supplement: Supplementary file 3 — Figure S2. Specialisation of LTR features on the whole dataset. (PPT 212 kb) [file 12864_2018_4901_MOESM3_ESM.ppt]

## Slide 1
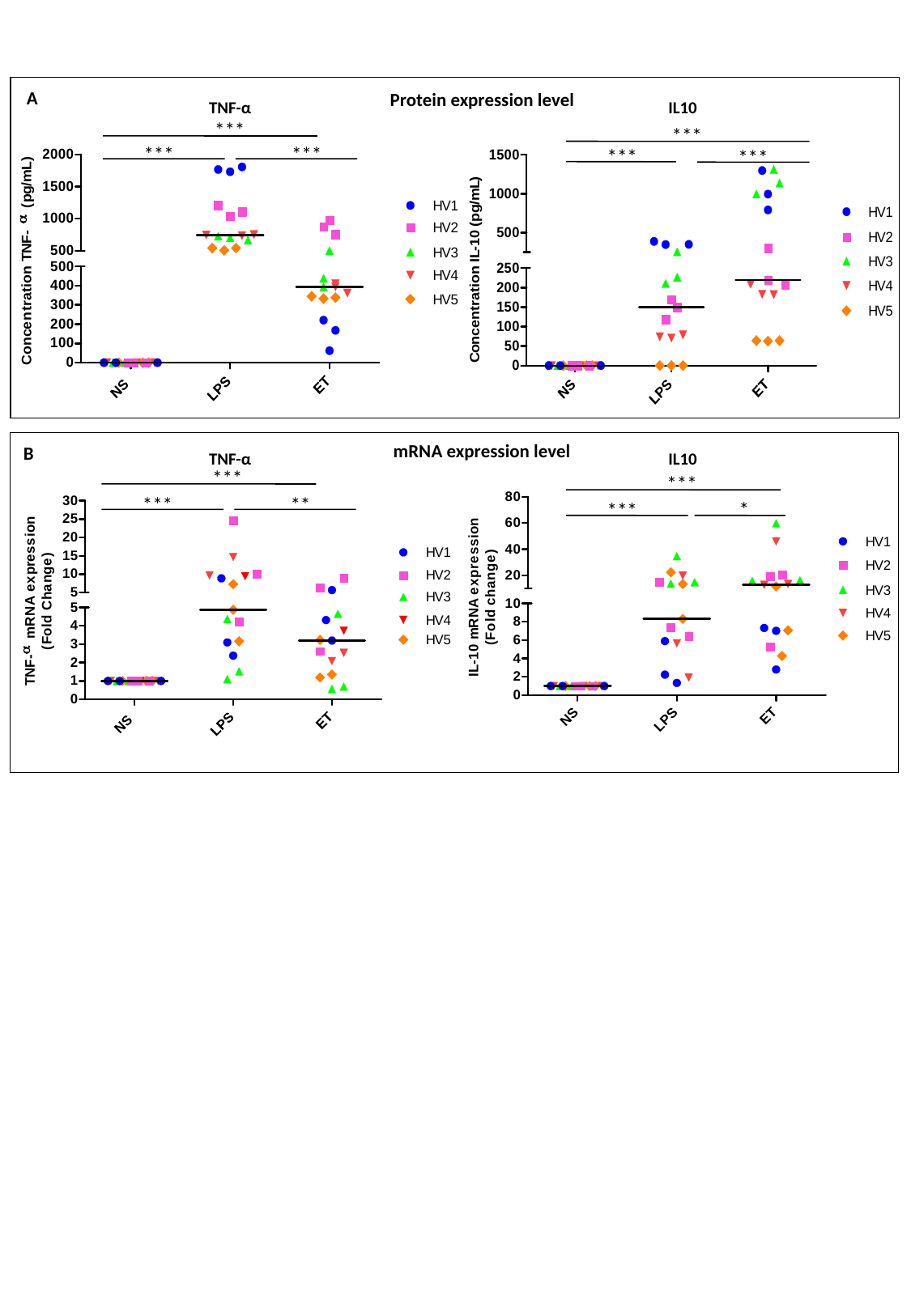

A
Protein expression level
TNF-α
IL10
***
***
***
***
***
***
mRNA expression level
B
TNF-α
IL10
***
***
***
**
*
***

Supplement: Supplementary file 5 — Figure S4. TNF-α and IL-10 protein assay and mRNA quantitation in PBMCs following LPS stimulations. (PPT 172 kb) [file 12864_2018_4901_MOESM5_ESM.ppt]
