# Supplementary material for: LTR-retrotransposon transcriptome modulation in response to endotoxin-induced stress in PBMCs
Source: BMC Genomics. 2018 Jul 5;19:522. doi: 10.1186/s12864-018-4901-9 (PMC6034278; doi:10.1186/s12864-018-4901-9)
Supplement: Supplementary file 4 — Figure S3. Genomic environment of functional and silent LTRs. (DOC 507 kb) [file 12864_2018_4901_MOESM4_ESM.doc]

**A**

| LTR function | nb of LTR | nb of neighbouring coding genes  (+/-50kb) | Ratio gene/LTR | Intragenic LTR orientation | |  | Intragenic LTR  location | | | | Intergenic LTR |
| --- | --- | --- | --- | --- | --- | --- | --- | --- | --- | --- | --- |
|  |  |  |  | sense | antisense |  | 5’UTR | Exon | Intron | 3’UTR |  |
| Pr | 297 | 207 | 0.7 | 43 | 76 |  | 9 | 15 | 113 | 10 | 178 |
| pA | 232 | 145 | 0.63 | 29 | 71 |  | 7 | 14 | 96 | 6 | 132 |
| No-Pr | 700 | 453 | 0.65 | 97 | 179 |  | 10 | 21 | 269 | 8 | 424 |
| No-pA | 484 | 319 | 0.66 | 76 | 124 |  | 23 | 14 | 191 | 10 | 284 |
| No | 1874 | 1026 | 0.55 | 262 | 409 |  | 31 | 58 | 655 | 24 | 1203 |

**B**

Genomic environment for 602 intergenic promoter LTRs


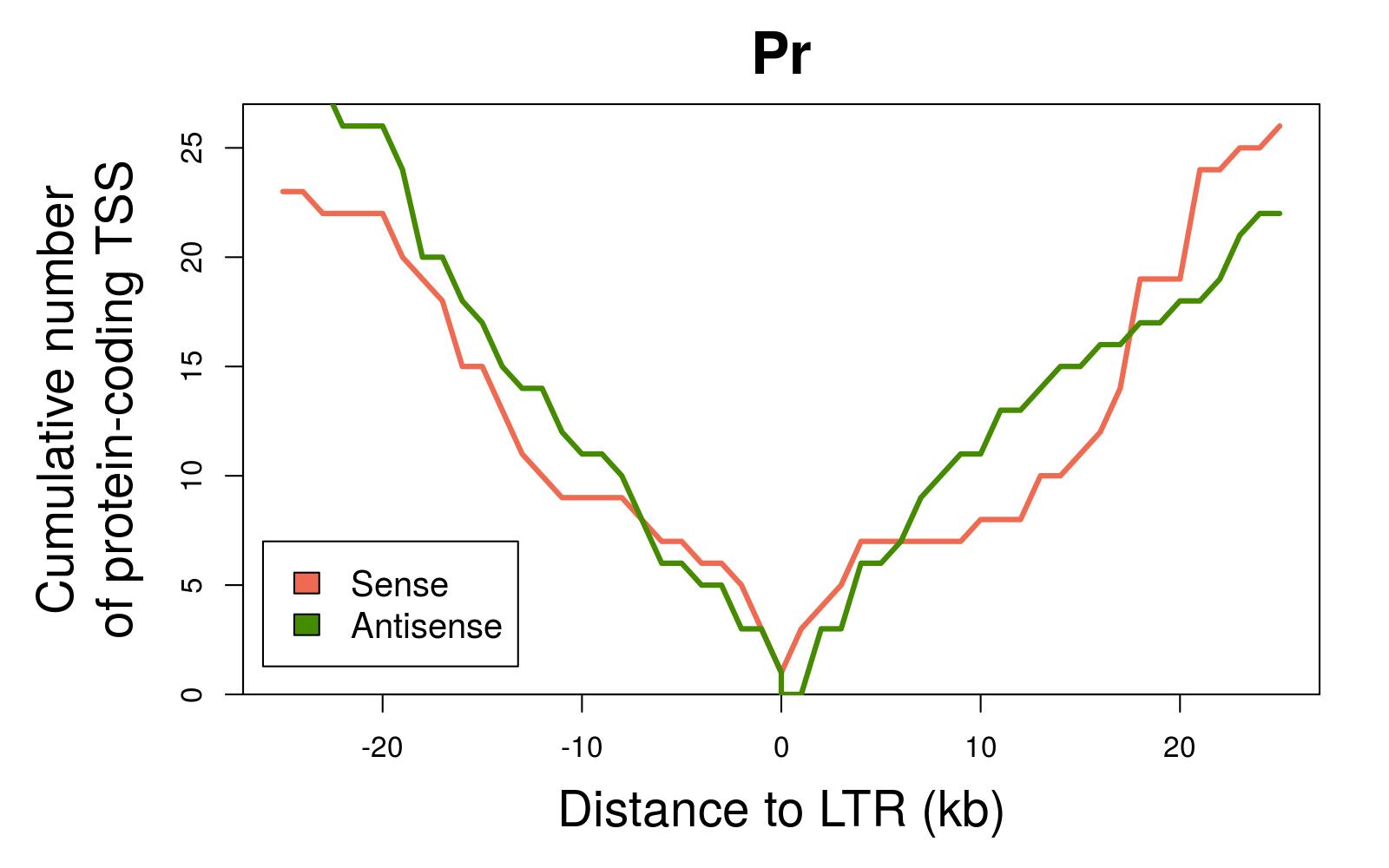

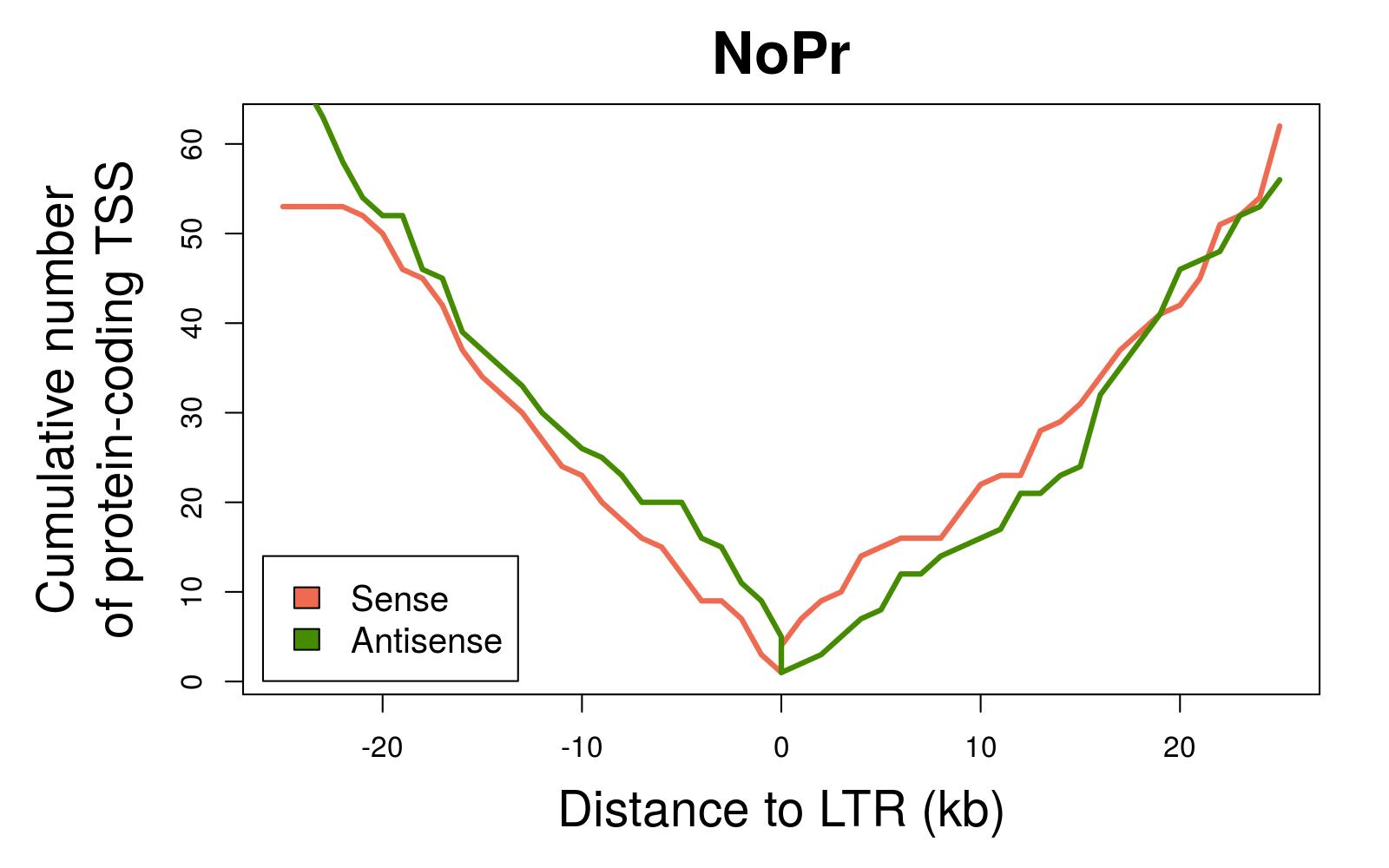


Genomic environment for 416 intergenic polyA LTRs


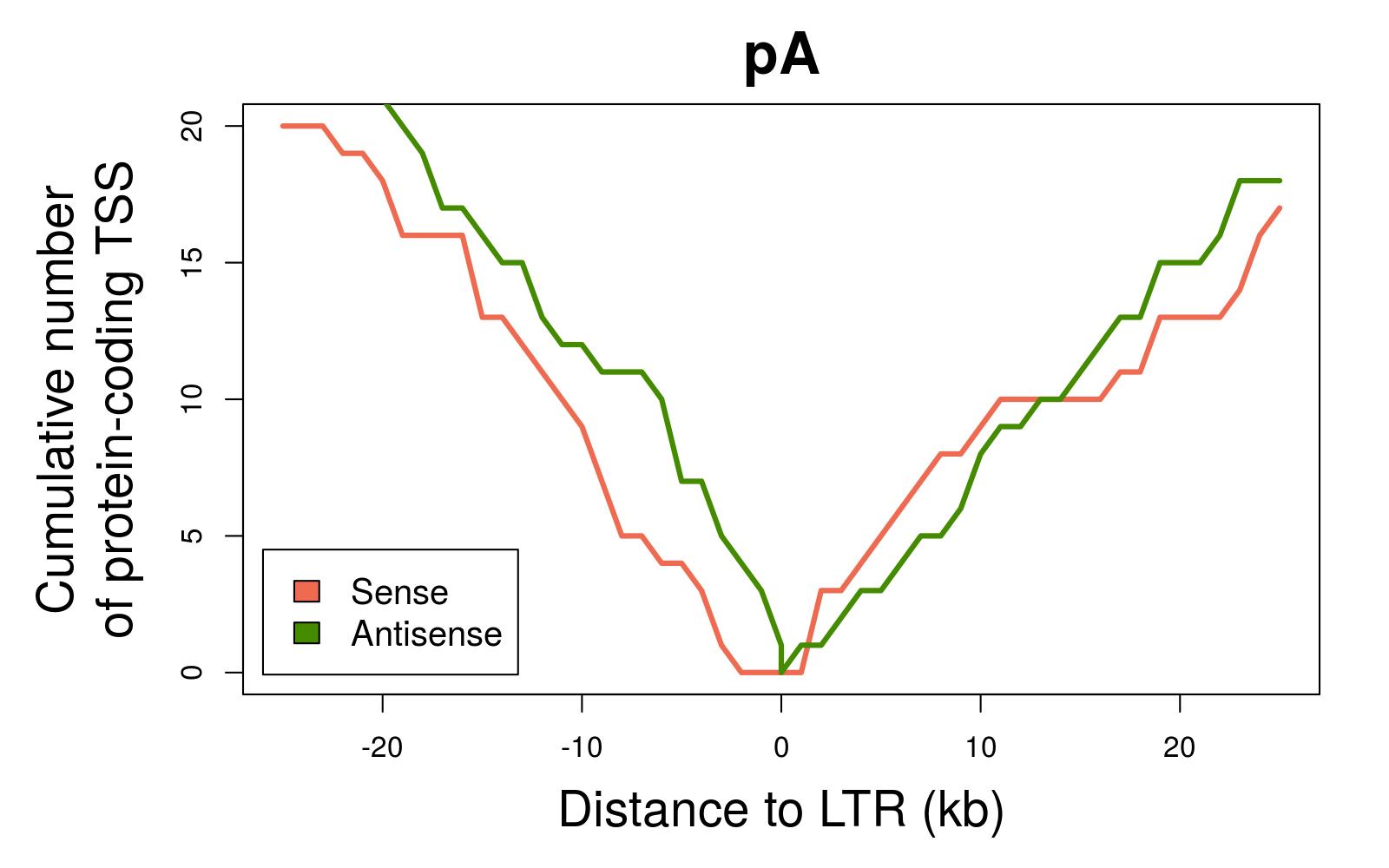

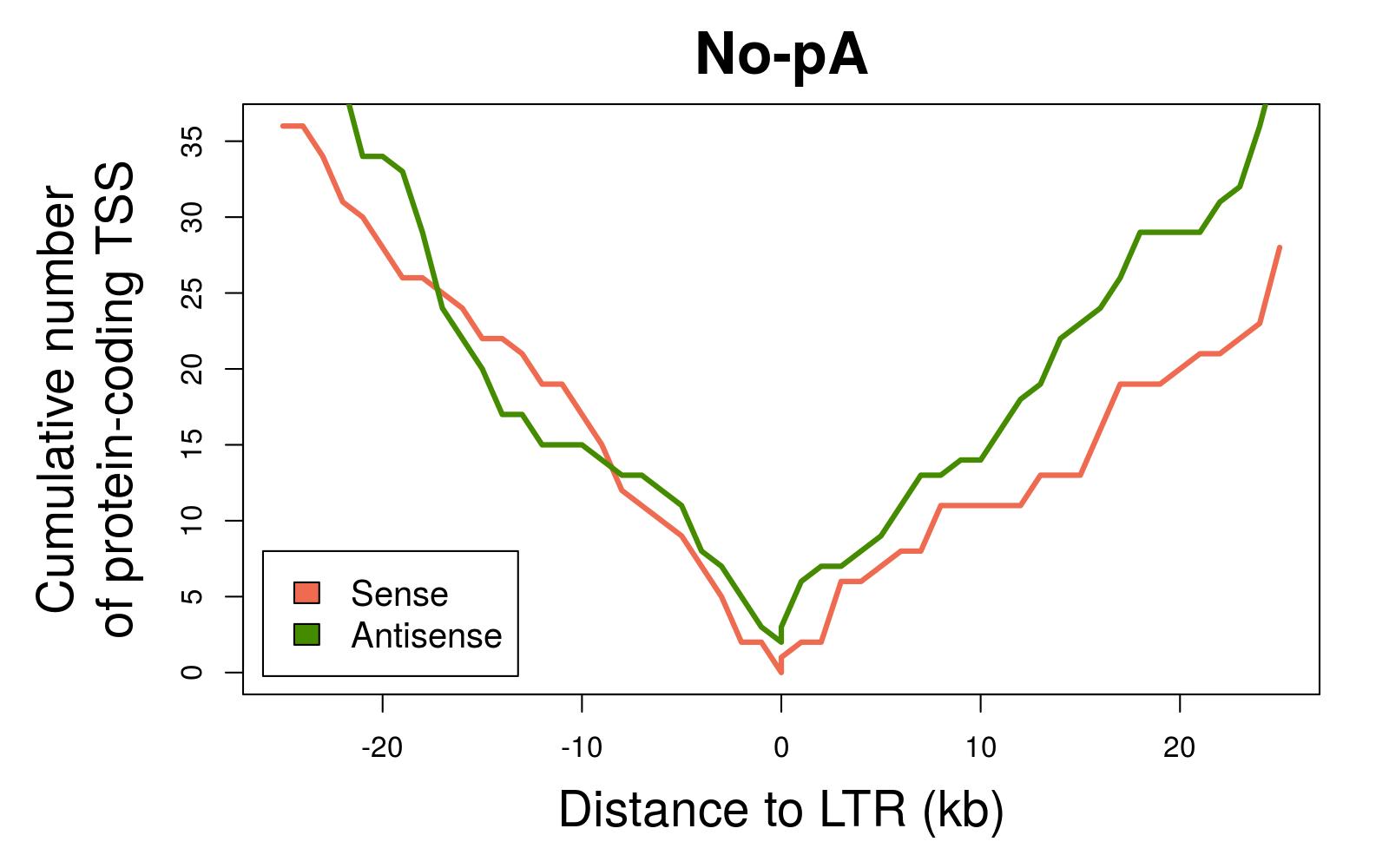


Genomic environment for 1203 intergenic silent LTRs

**
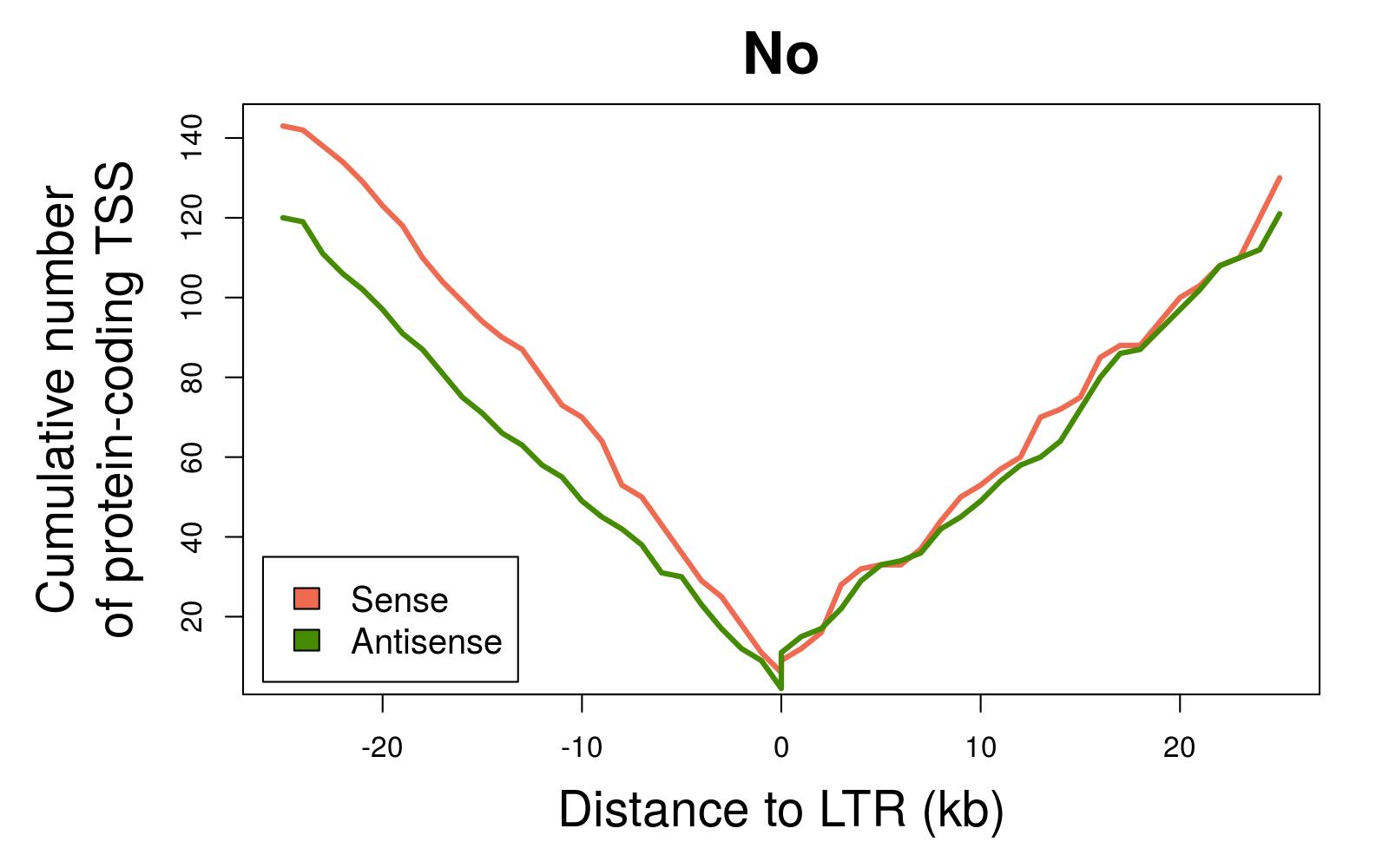
**
