# Supplementary material for: LTR-retrotransposon transcriptome modulation in response to endotoxin-induced stress in PBMCs
Source: BMC Genomics. 2018 Jul 5;19:522. doi: 10.1186/s12864-018-4901-9 (PMC6034278; doi:10.1186/s12864-018-4901-9)
Supplement: Supplementary file 7 — Figure S5. Selection, design and quality criteria for the design of locus specific qPCR systems, illustrated with the 121601901-HERV0116uL locus, and PCR systems obtained. (PPT 766 kb) [file 12864_2018_4901_MOESM7_ESM.ppt]

## Slide 1
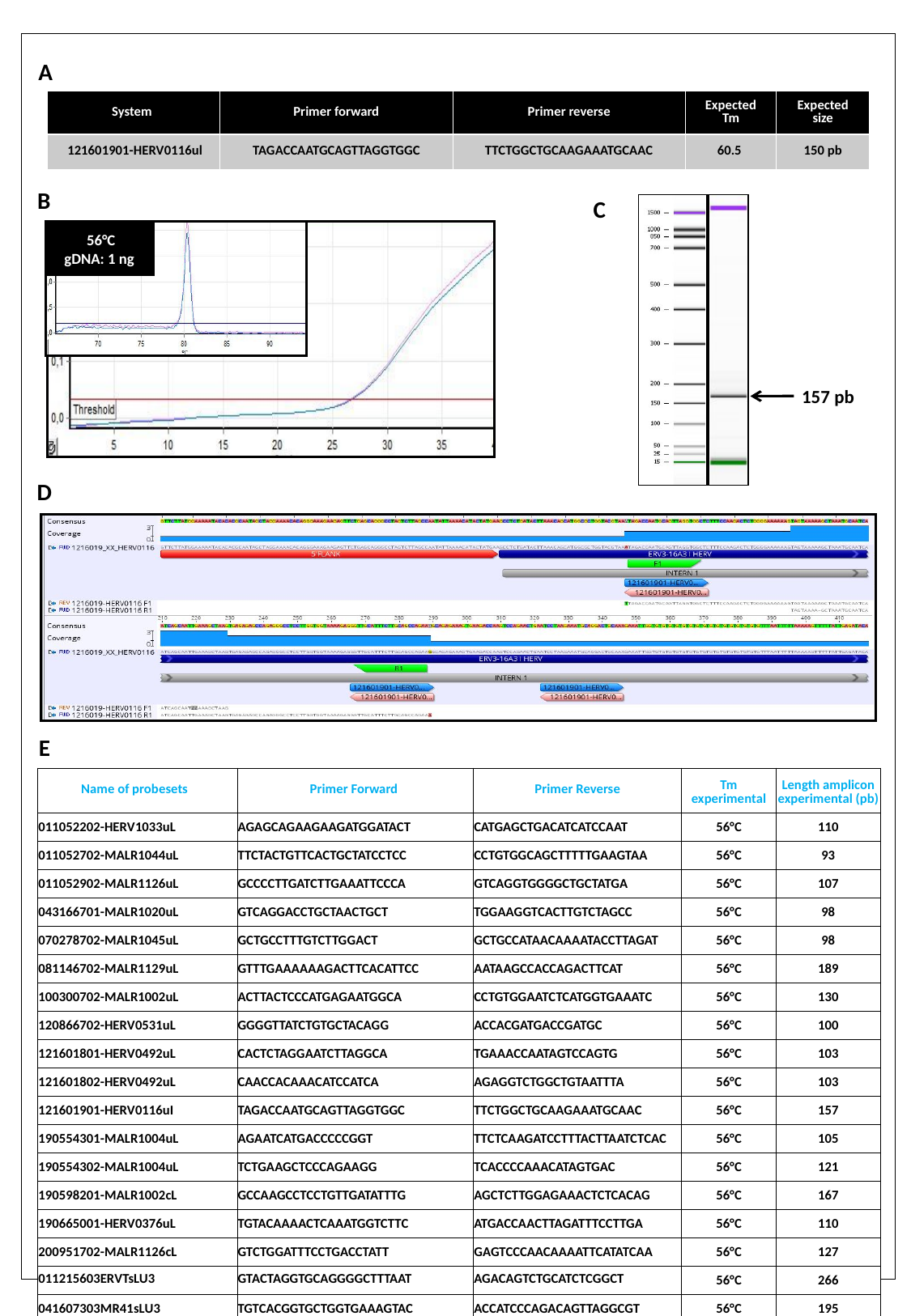

A
| System | Primer forward | Primer reverse | Expected Tm | Expected size |
| --- | --- | --- | --- | --- |
| 121601901-HERV0116ul | TAGACCAATGCAGTTAGGTGGC | TTCTGGCTGCAAGAAATGCAAC | 60.5 | 150 pb |
B
C
56°C
gDNA: 1 ng
157 pb
D
E
| Name of probesets | Primer Forward | Primer Reverse | Tm experimental | Length amplicon experimental (pb) |
| --- | --- | --- | --- | --- |
| 011052202-HERV1033uL | AGAGCAGAAGAAGATGGATACT | CATGAGCTGACATCATCCAAT | 56°C | 110 |
| 011052702-MALR1044uL | TTCTACTGTTCACTGCTATCCTCC | CCTGTGGCAGCTTTTTGAAGTAA | 56°C | 93 |
| 011052902-MALR1126uL | GCCCCTTGATCTTGAAATTCCCA | GTCAGGTGGGGCTGCTATGA | 56°C | 107 |
| 043166701-MALR1020uL | GTCAGGACCTGCTAACTGCT | TGGAAGGTCACTTGTCTAGCC | 56°C | 98 |
| 070278702-MALR1045uL | GCTGCCTTTGTCTTGGACT | GCTGCCATAACAAAATACCTTAGAT | 56°C | 98 |
| 081146702-MALR1129uL | GTTTGAAAAAAGACTTCACATTCC | AATAAGCCACCAGACTTCAT | 56°C | 189 |
| 100300702-MALR1002uL | ACTTACTCCCATGAGAATGGCA | CCTGTGGAATCTCATGGTGAAATC | 56°C | 130 |
| 120866702-HERV0531uL | GGGGTTATCTGTGCTACAGG | ACCACGATGACCGATGC | 56°C | 100 |
| 121601801-HERV0492uL | CACTCTAGGAATCTTAGGCA | TGAAACCAATAGTCCAGTG | 56°C | 103 |
| 121601802-HERV0492uL | CAACCACAAACATCCATCA | AGAGGTCTGGCTGTAATTTA | 56°C | 103 |
| 121601901-HERV0116uI | TAGACCAATGCAGTTAGGTGGC | TTCTGGCTGCAAGAAATGCAAC | 56°C | 157 |
| 190554301-MALR1004uL | AGAATCATGACCCCCGGT | TTCTCAAGATCCTTTACTTAATCTCAC | 56°C | 105 |
| 190554302-MALR1004uL | TCTGAAGCTCCCAGAAGG | TCACCCCAAACATAGTGAC | 56°C | 121 |
| 190598201-MALR1002cL | GCCAAGCCTCCTGTTGATATTTG | AGCTCTTGGAGAAACTCTCACAG | 56°C | 167 |
| 190665001-HERV0376uL | TGTACAAAACTCAAATGGTCTTC | ATGACCAACTTAGATTTCCTTGA | 56°C | 110 |
| 200951702-MALR1126cL | GTCTGGATTTCCTGACCTATT | GAGTCCCAACAAAATTCATATCAA | 56°C | 127 |
| 011215603ERVTsLU3 | GTACTAGGTGCAGGGGCTTTAAT | AGACAGTCTGCATCTCGGCT | 56°C | 266 |
| 041607303MR41sLU3 | TGTCACGGTGCTGGTGAAAGTAC | ACCATCCCAGACAGTTAGGCGT | 56°C | 195 |
| 052539003PBLAsLU3p | CTTTAGGCCCCCAATAAACCTG | TAGGTCTCAGTTATGCAGGCTT | 56°C | 260 |
| 100082202HK063LRp | AGTTTCTACAGTCTTTCATATTTGTTAG | GACCTGTGATAAATCAAGCAGTG | 56°C | 97 |
| | | | | |
| 020145402-MALR1127uL | AGAACACCAGTCATATTGGATTTG | CGCTTTGGAATGGGACTGTT | 56°C | 118 |
| 091606902-MALR1005cL | GAAACCAACTCGCCAATACC | CAGGACCTGTGAGTATGTGAC | 56°C | 166 |
| 100248101-MALR1126uL | GGCTGAATTCCATCCTAAC | CAACAGTCATATTGGATTAAGAT | 56°C | 130 |

## Slide 2
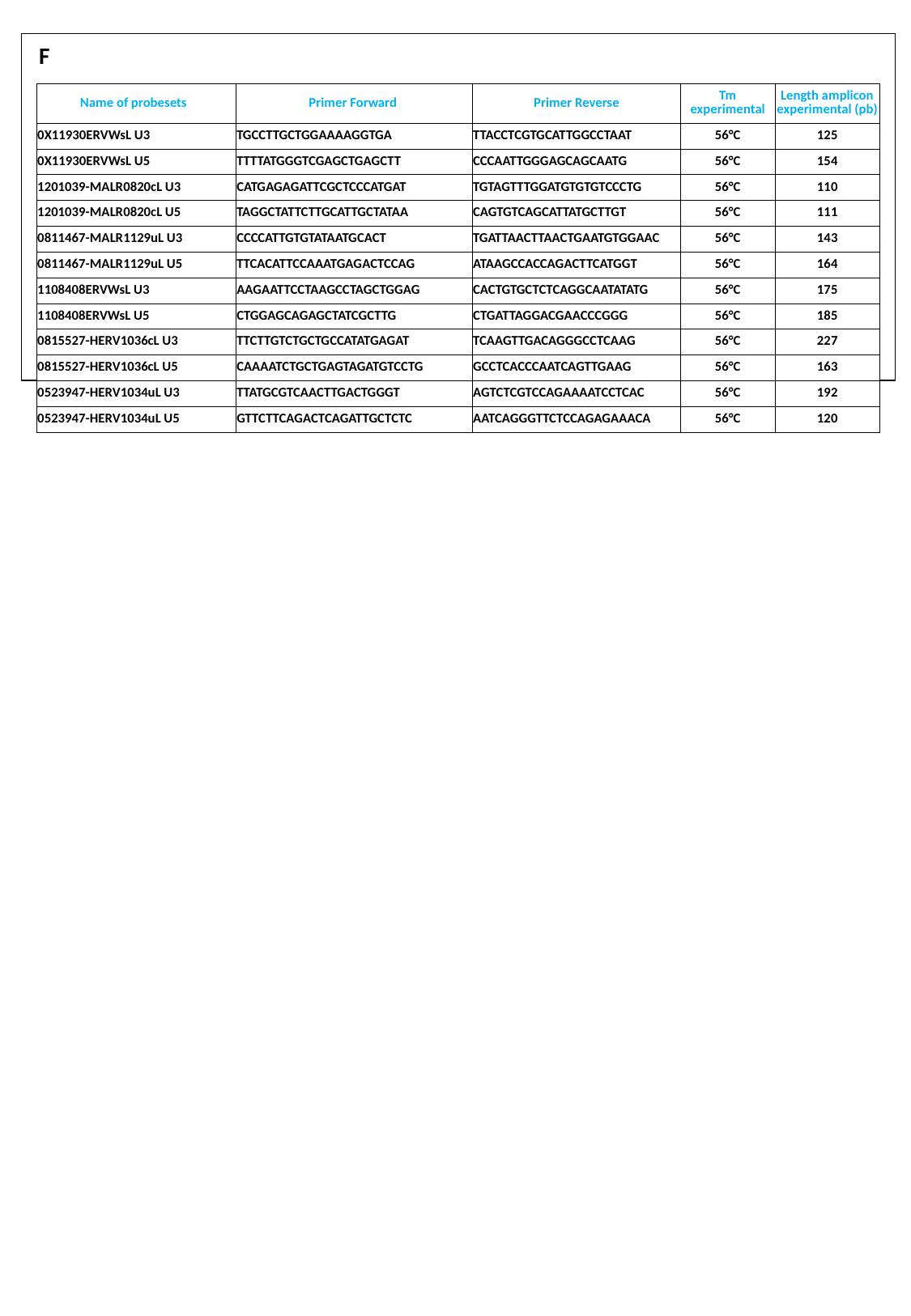

F
| Name of probesets | Primer Forward | Primer Reverse | Tm experimental | Length amplicon experimental (pb) |
| --- | --- | --- | --- | --- |
| 0X11930ERVWsL U3 | TGCCTTGCTGGAAAAGGTGA | TTACCTCGTGCATTGGCCTAAT | 56°C | 125 |
| 0X11930ERVWsL U5 | TTTTATGGGTCGAGCTGAGCTT | CCCAATTGGGAGCAGCAATG | 56°C | 154 |
| 1201039-MALR0820cL U3 | CATGAGAGATTCGCTCCCATGAT | TGTAGTTTGGATGTGTGTCCCTG | 56°C | 110 |
| 1201039-MALR0820cL U5 | TAGGCTATTCTTGCATTGCTATAA | CAGTGTCAGCATTATGCTTGT | 56°C | 111 |
| 0811467-MALR1129uL U3 | CCCCATTGTGTATAATGCACT | TGATTAACTTAACTGAATGTGGAAC | 56°C | 143 |
| 0811467-MALR1129uL U5 | TTCACATTCCAAATGAGACTCCAG | ATAAGCCACCAGACTTCATGGT | 56°C | 164 |
| 1108408ERVWsL U3 | AAGAATTCCTAAGCCTAGCTGGAG | CACTGTGCTCTCAGGCAATATATG | 56°C | 175 |
| 1108408ERVWsL U5 | CTGGAGCAGAGCTATCGCTTG | CTGATTAGGACGAACCCGGG | 56°C | 185 |
| 0815527-HERV1036cL U3 | TTCTTGTCTGCTGCCATATGAGAT | TCAAGTTGACAGGGCCTCAAG | 56°C | 227 |
| 0815527-HERV1036cL U5 | CAAAATCTGCTGAGTAGATGTCCTG | GCCTCACCCAATCAGTTGAAG | 56°C | 163 |
| 0523947-HERV1034uL U3 | TTATGCGTCAACTTGACTGGGT | AGTCTCGTCCAGAAAATCCTCAC | 56°C | 192 |
| 0523947-HERV1034uL U5 | GTTCTTCAGACTCAGATTGCTCTC | AATCAGGGTTCTCCAGAGAAACA | 56°C | 120 |
