# Supplementary material for: LTR-retrotransposon transcriptome modulation in response to endotoxin-induced stress in PBMCs
Source: BMC Genomics. 2018 Jul 5;19:522. doi: 10.1186/s12864-018-4901-9 (PMC6034278; doi:10.1186/s12864-018-4901-9)
Supplement: Supplementary file 8 — Figure S6. RT-qPCR validation of 23 microarray-based identified HERV/MaLR elements differentially expressed following LPS stimulations. (PPT 1309 kb) [file 12864_2018_4901_MOESM8_ESM.ppt]

## Slide 1
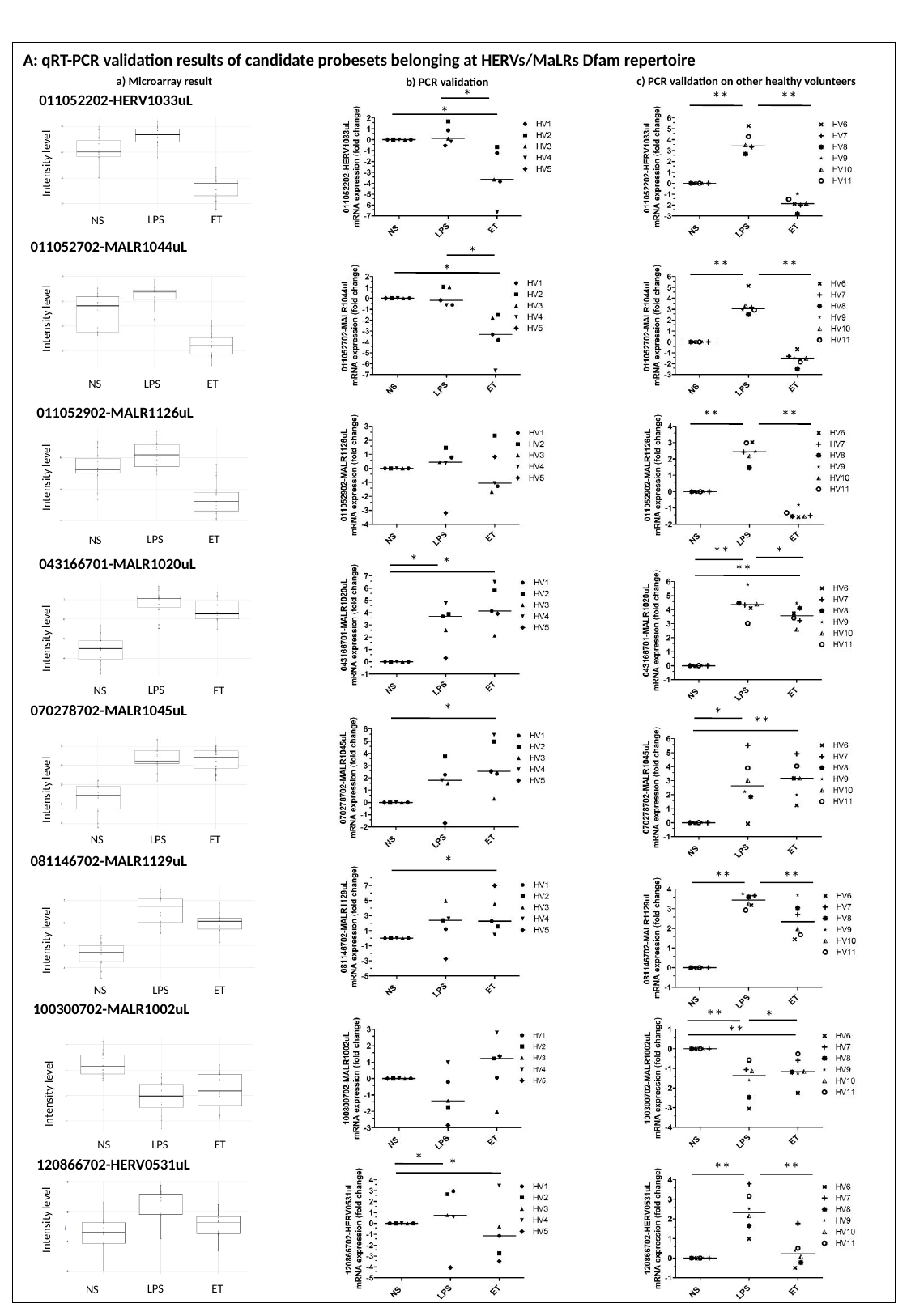

A: qRT-PCR validation results of candidate probesets belonging at HERVs/MaLRs Dfam repertoire
a) Microarray result
c) PCR validation on other healthy volunteers
b) PCR validation
*
**
**
011052202-HERV1033uL
*
 Intensity level
 LPS
 ET
 NS
011052702-MALR1044uL
*
**
**
*
 Intensity level
 NS
 LPS
 ET
011052902-MALR1126uL
**
**
 Intensity level
 LPS
 ET
 NS
**
*
*
*
043166701-MALR1020uL
**
 Intensity level
 LPS
 ET
 NS
*
070278702-MALR1045uL
*
**
 Intensity level
 NS
 LPS
 ET
081146702-MALR1129uL
*
**
**
 Intensity level
 NS
 LPS
 ET
100300702-MALR1002uL
**
*
**
 Intensity level
 NS
 LPS
 ET
*
*
120866702-HERV0531uL
**
**
 Intensity level
 LPS
 ET
 NS

## Slide 2
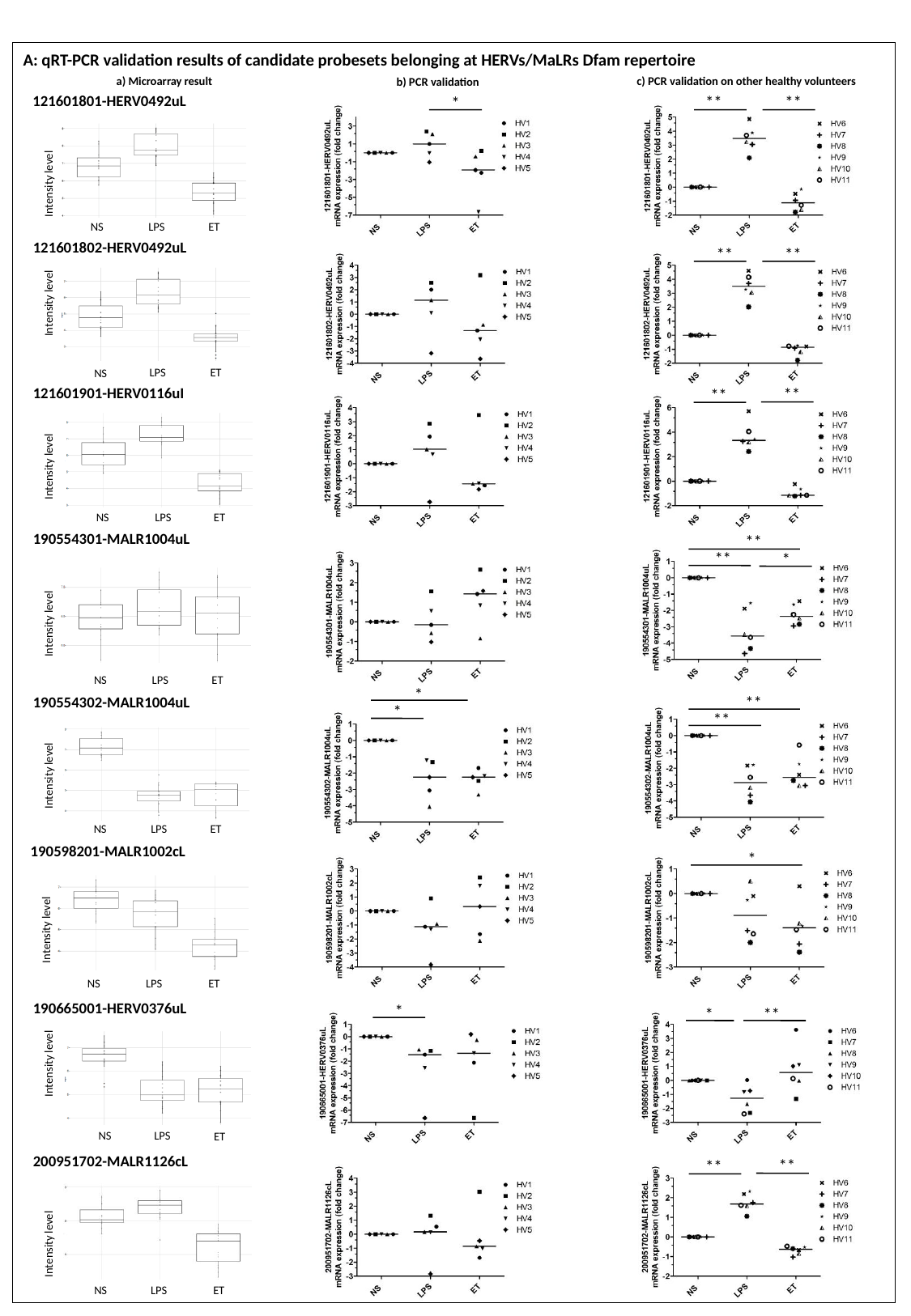

A: qRT-PCR validation results of candidate probesets belonging at HERVs/MaLRs Dfam repertoire
a) Microarray result
c) PCR validation on other healthy volunteers
b) PCR validation
121601801-HERV0492uL
**
**
*
 Intensity level
 NS
 LPS
 ET
121601802-HERV0492uL
**
**
 Intensity level
 LPS
 ET
 NS
121601901-HERV0116uI
**
**
 Intensity level
 NS
 LPS
 ET
190554301-MALR1004uL
**
**
*
 Intensity level
 NS
 LPS
 ET
*
**
190554302-MALR1004uL
*
**
 Intensity level
 NS
 LPS
 ET
190598201-MALR1002cL
*
 Intensity level
 NS
 LPS
 ET
190665001-HERV0376uL
*
**
*
 Intensity level
 NS
 LPS
 ET
200951702-MALR1126cL
**
**
 Intensity level
 NS
 LPS
 ET

## Slide 3
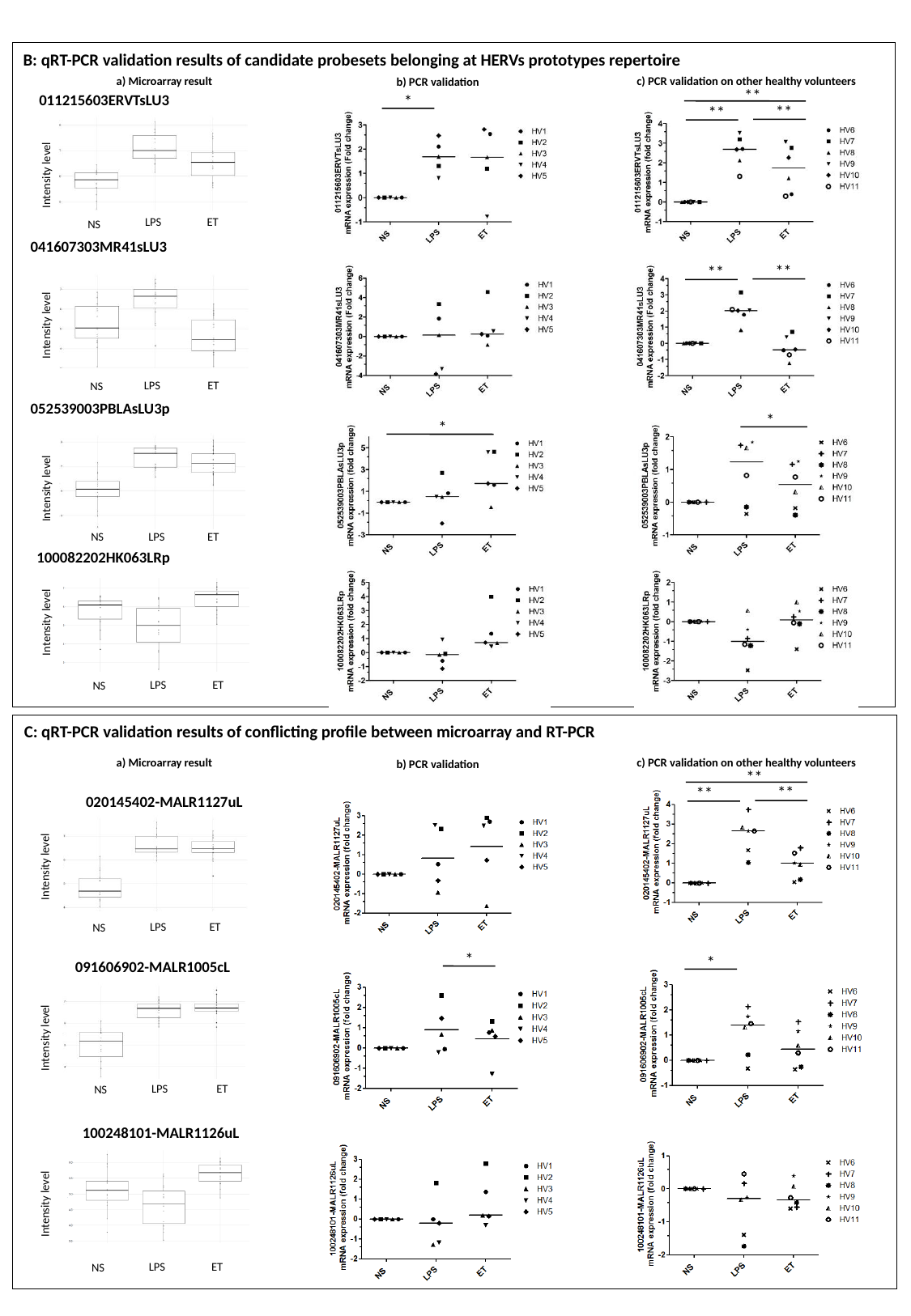

B: qRT-PCR validation results of candidate probesets belonging at HERVs prototypes repertoire
a) Microarray result
c) PCR validation on other healthy volunteers
b) PCR validation
**
011215603ERVTsLU3
*
**
**
 Intensity level
 ET
 LPS
 NS
041607303MR41sLU3
**
**
 Intensity level
 LPS
 ET
 NS
052539003PBLAsLU3p
*
*
 Intensity level
 NS
 LPS
 ET
100082202HK063LRp
 Intensity level
 LPS
 ET
 NS
C: qRT-PCR validation results of conflicting profile between microarray and RT-PCR
a) Microarray result
c) PCR validation on other healthy volunteers
b) PCR validation
**
**
**
020145402-MALR1127uL
 Intensity level
 LPS
 ET
 NS
*
*
091606902-MALR1005cL
 Intensity level
 LPS
 ET
 NS
100248101-MALR1126uL
 Intensity level
 LPS
 ET
 NS

## Slide 4
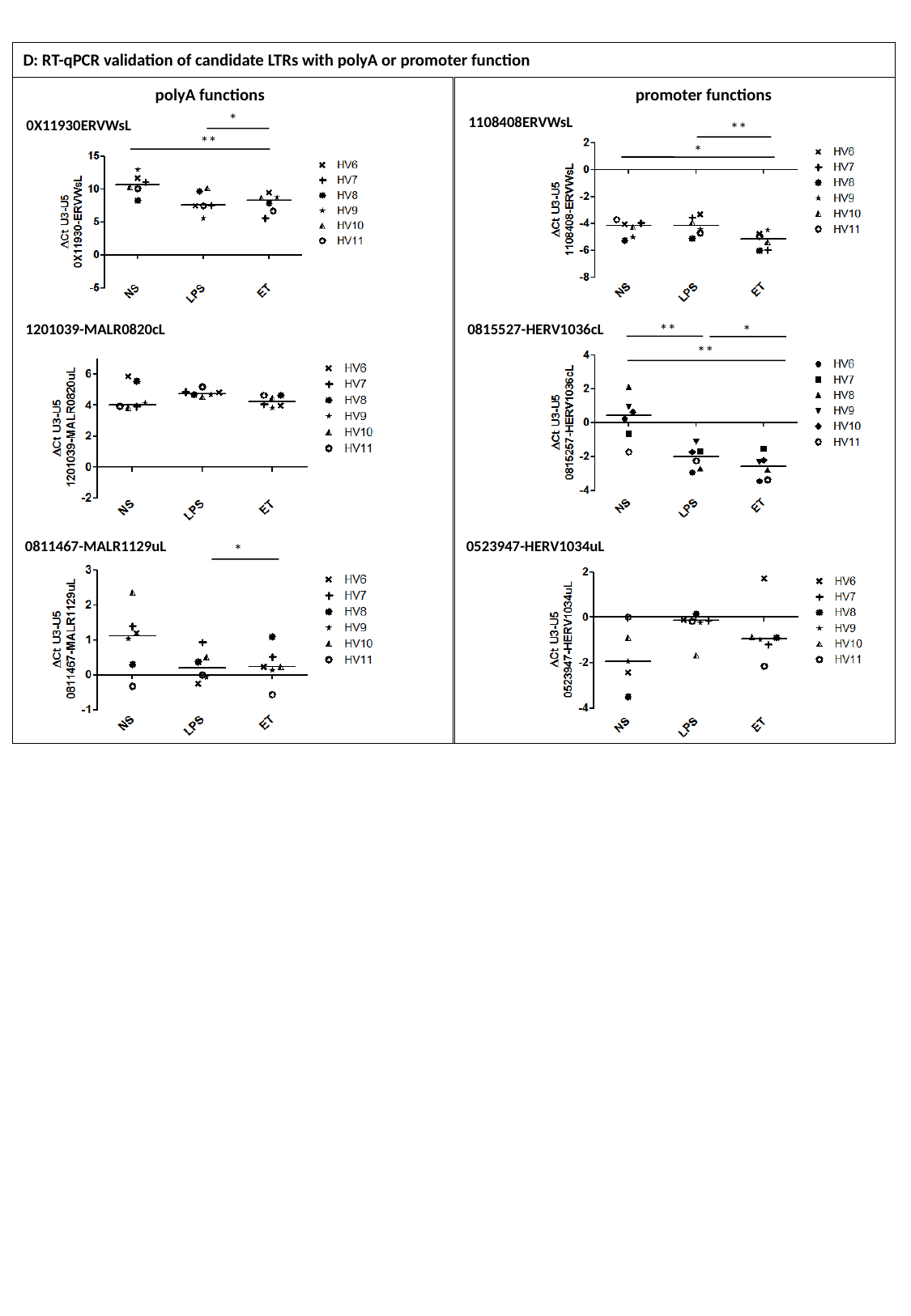

D: RT-qPCR validation of candidate LTRs with polyA or promoter function
polyA functions
promoter functions
*
1108408ERVWsL
0X11930ERVWsL
**
**
*
0815527-HERV1036cL
1201039-MALR0820cL
**
*
**
0811467-MALR1129uL
0523947-HERV1034uL
*
