# Supplementary material for: LTR-retrotransposon transcriptome modulation in response to endotoxin-induced stress in PBMCs
Source: BMC Genomics. 2018 Jul 5;19:522. doi: 10.1186/s12864-018-4901-9 (PMC6034278; doi:10.1186/s12864-018-4901-9)
Supplement: Supplementary file 9 — Figure S7. Strategy used to integrate 62 HERVs/MaLRs and 26 genes in 11 canonical immune pathways, global landscape resulting from this analysis, and identification of PTGS2 and FLT1 associated HERVs/MaLRs at the crossroads of “LXR/RXR activation” and “NF-kB signalling” pathways. (PPT 1323 kb) [file 12864_2018_4901_MOESM9_ESM.ppt]

## Slide 1
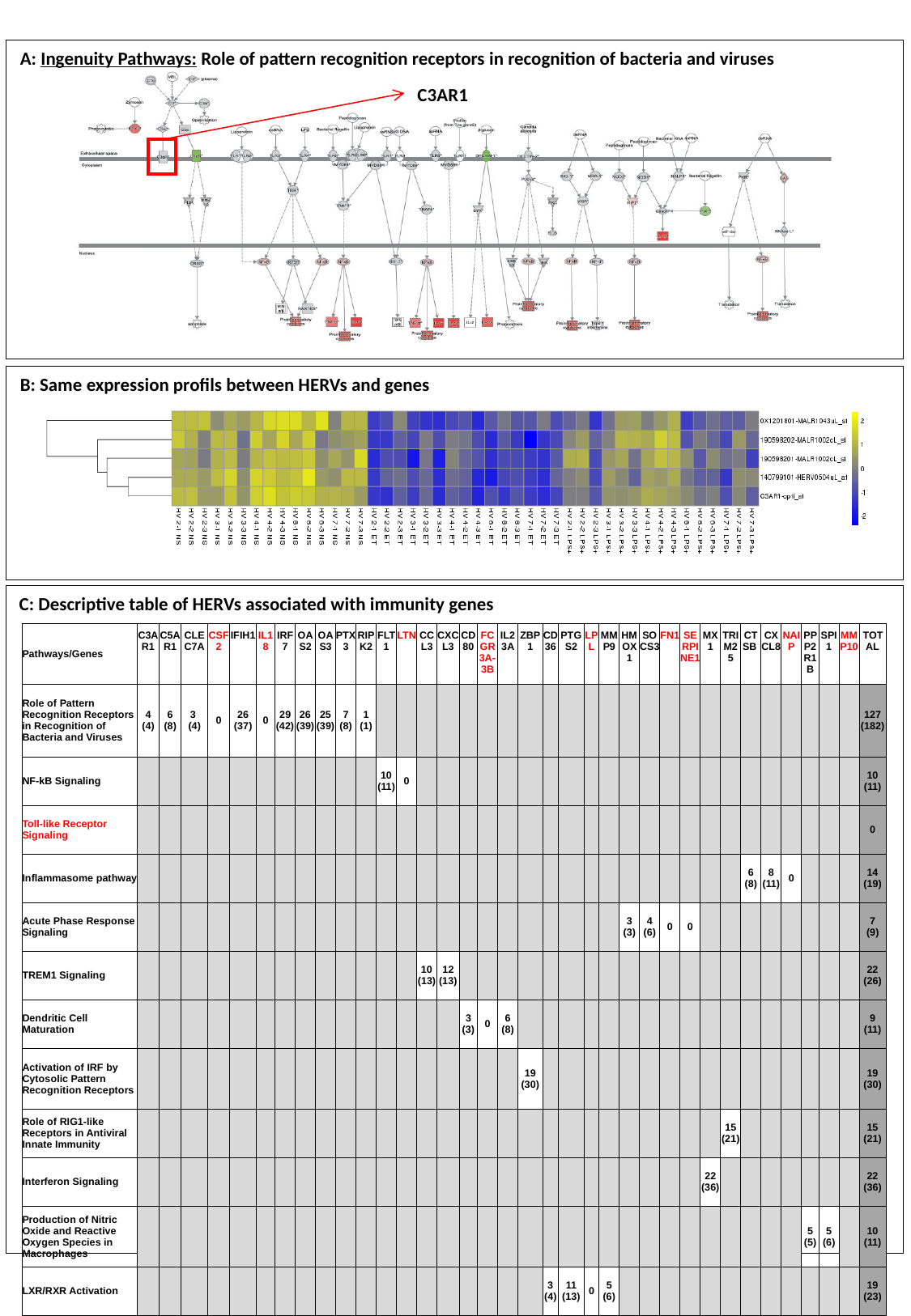

A: Ingenuity Pathways: Role of pattern recognition receptors in recognition of bacteria and viruses
C3AR1
B: Same expression profils between HERVs and genes
C: Descriptive table of HERVs associated with immunity genes
| Pathways/Genes | C3AR1 | C5AR1 | CLEC7A | CSF2 | IFIH1 | IL18 | IRF7 | OAS2 | OAS3 | PTX3 | RIPK2 | FLT1 | LTN | CCL3 | CXCL3 | CD80 | FCGR3A-3B | IL23A | ZBP1 | CD36 | PTGS2 | LPL | MMP9 | HMOX1 | SOCS3 | FN1 | SERPINE1 | MX1 | TRIM25 | CTSB | CXCL8 | NAIP | PPP2R1B | SPI1 | MMP10 | TOTAL |
| --- | --- | --- | --- | --- | --- | --- | --- | --- | --- | --- | --- | --- | --- | --- | --- | --- | --- | --- | --- | --- | --- | --- | --- | --- | --- | --- | --- | --- | --- | --- | --- | --- | --- | --- | --- | --- |
| Role of Pattern Recognition Receptors in Recognition of Bacteria and Viruses | 4 (4) | 6 (8) | 3 (4) | 0 | 26 (37) | 0 | 29 (42) | 26 (39) | 25 (39) | 7 (8) | 1 (1) | | | | | | | | | | | | | | | | | | | | | | | | | 127 (182) |
| NF-kB Signaling | | | | | | | | | | | | 10 (11) | 0 | | | | | | | | | | | | | | | | | | | | | | | 10 (11) |
| Toll-like Receptor Signaling | | | | | | | | | | | | | | | | | | | | | | | | | | | | | | | | | | | | 0 |
| Inflammasome pathway | | | | | | | | | | | | | | | | | | | | | | | | | | | | | | 6 (8) | 8 (11) | 0 | | | | 14 (19) |
| Acute Phase Response Signaling | | | | | | | | | | | | | | | | | | | | | | | | 3 (3) | 4 (6) | 0 | 0 | | | | | | | | | 7 (9) |
| TREM1 Signaling | | | | | | | | | | | | | | 10 (13) | 12 (13) | | | | | | | | | | | | | | | | | | | | | 22 (26) |
| Dendritic Cell Maturation | | | | | | | | | | | | | | | | 3 (3) | 0 | 6 (8) | | | | | | | | | | | | | | | | | | 9 (11) |
| Activation of IRF by Cytosolic Pattern Recognition Receptors | | | | | | | | | | | | | | | | | | | 19 (30) | | | | | | | | | | | | | | | | | 19 (30) |
| Role of RIG1-like Receptors in Antiviral Innate Immunity | | | | | | | | | | | | | | | | | | | | | | | | | | | | | 15 (21) | | | | | | | 15 (21) |
| Interferon Signaling | | | | | | | | | | | | | | | | | | | | | | | | | | | | 22 (36) | | | | | | | | 22 (36) |
| Production of Nitric Oxide and Reactive Oxygen Species in Macrophages | | | | | | | | | | | | | | | | | | | | | | | | | | | | | | | | | 5 (5) | 5 (6) | | 10 (11) |
| LXR/RXR Activation | | | | | | | | | | | | | | | | | | | | 3 (4) | 11 (13) | 0 | 5 (6) | | | | | | | | | | | | | 19 (23) |
| Colorectal Cancer Metastasis Signaling | | | | | | | | | | | | | | | | | | | | | | | | | | | | | | | | | | | 0 | |

## Slide 2
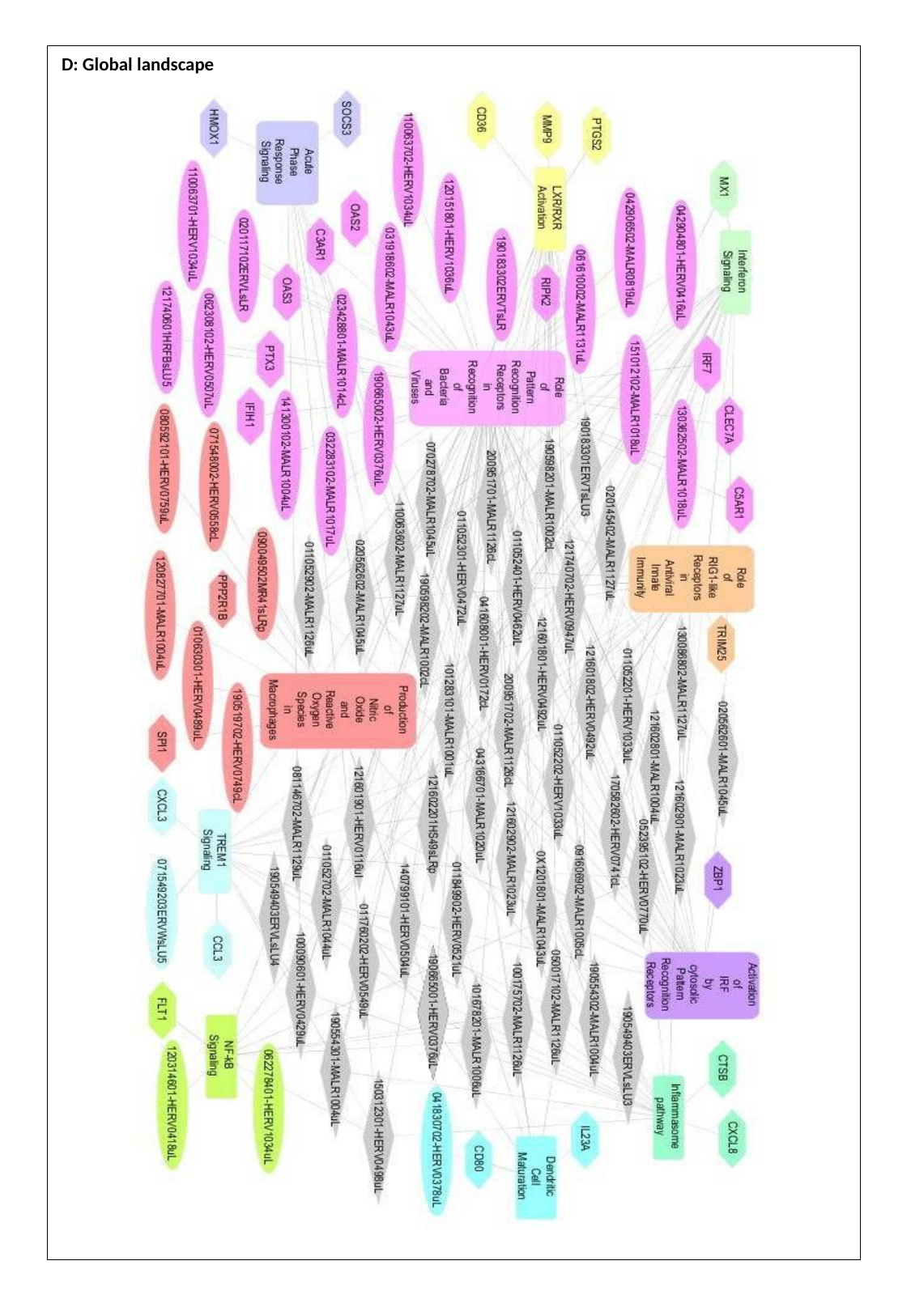

D: Global landscape

## Slide 3
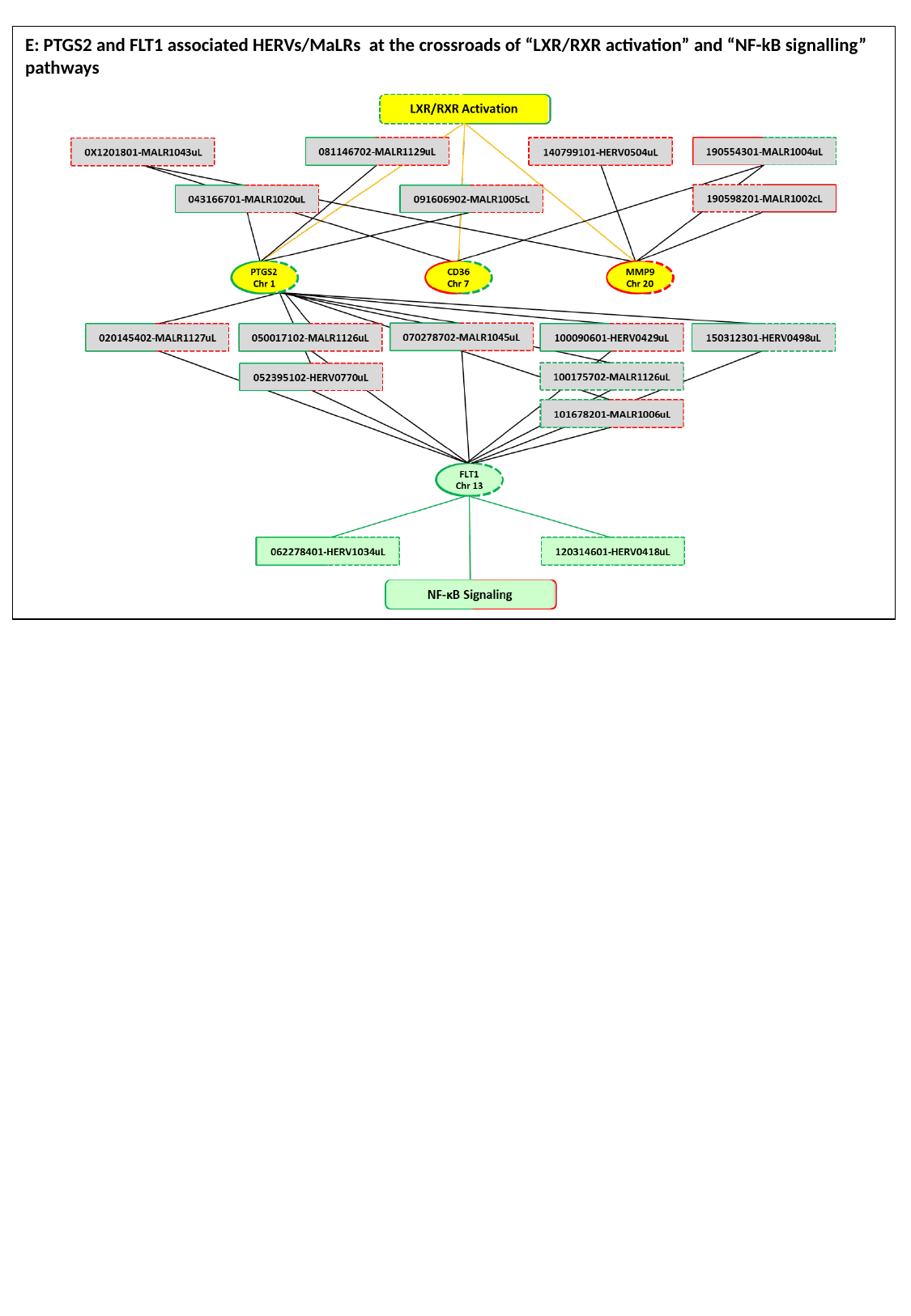

E: PTGS2 and FLT1 associated HERVs/MaLRs at the crossroads of “LXR/RXR activation” and “NF-kB signalling” pathways
