# Supplementary material for: LTR-retrotransposon transcriptome modulation in response to endotoxin-induced stress in PBMCs
Source: BMC Genomics. 2018 Jul 5;19:522. doi: 10.1186/s12864-018-4901-9 (PMC6034278; doi:10.1186/s12864-018-4901-9)
Supplement: Supplementary file 11 — Supplementary Methods. (DOC 55 kb) [file 12864_2018_4901_MOESM11_ESM.doc]

**Supplementary Methods:**

*Microarray Analysis Pre-processing*

CEL files were transformed into a matrix, normalised, adjusted for background noise (RMA normalisation), and probes were summarised into probesets with the apt-probeset-summarize command (V 1.18.0) with the rma option. Microarray pre-processing and statistical analysis were performed using R/Bioconductor (R v3.1.2) . Quality assessment was performed using simpleaffy (v2.42.0) and arrayQualityMetrics (v3.22.1) . For quality control, several criteria were used: RNA quality, images of chips, hybridisation spike-in, PolyA, Amplification and fragmentation, intensity signals (before and after normalisation), probeset homogeneity (RLE, NUSE plots), correlation plots (before and after normalization) and Principal Component Analysis. For most criteria, outlier detection was performed by computing the Kolmogorov Smirnof (KS) statistic between each array and the pooled data (default threshold with arrayQualityMetrics library). An array was removed from the analysis if it did not pass more than four quality controls. No array was removed based on these criteria. The data were then normalised, adjusted for background noise and summarised using the RMA (Robust Multi-Array) algorithm . Experiment batch effects were removed using COMBAT . Finally, a filtering process was performed to reduce the dataset and gain statistical power for analyses. A Coefficient of Variation (CV) value of 10% was used to determine the intensity threshold from which the 3^rd^ quartile of intensity ranked by range of values is under the CV value. Probesets under the intensity threshold in more than 68% of all samples (14 samples out of 45) were then removed. Consequently, a probeset was counted as transcriptionally active if the normalised signal was observed (i) above a 2^5.5^ threshold , corresponding to the minimum intensity level shared by all repertoires exhibiting an acceptable variability (defined as the value of the 75th percentile of the distribution of the coefficient of variation as a function of intensity lower than 10%) (Additional file 1: Figure S1), and (ii) for at least 14 samples out of 45.

*Design of integrative pathways*

The Ingenuity Pathways Analysis tool (IPA, Ingenuity® Systems, [www.ingenuity.com](http://www.ingenuity.com/)) was used to assess upstream regulators, canonical pathways, disease and functions. This analysis was performed using both the HTA and U133 gene repertoire, the significant pathway was extracted from the HTA gene repertoire because the result is similar to the U133 repertoire, but also more complete and informative than the U133 repertoire. Only canonical pathways were analysed between all conditions in the present study. Canonical pathways were predicted to be activated or inhibited for z-scores ≥ 2 and ≤ -2 respectively; a p-value cut-off of 0.05 was used (Fisher’s exact test). Predicted pathways between inflammatory and immunocompromised contexts were selected, and the genes present exclusively in one pathway were chosen for pathway analysis. For each single gene in the hierarchical clustering analysis, HERVs and MALRs or other elements with Pearson’s correlation at 0.8 are considered to be co-expressed with the gene. The global network is constructed with cytoscape software ([www.cytoscape.org](http://www.cytoscape.org/)).

*Primer Design and Validation*

PCR primers were designed where possible overlapping with probes that allowed their detection onto the chip. The design of locus-specific primer pairs was selected based on HERV-V3 microarray analysis using Primer3 and NCBI Primer-BLAST software ([www.ncbi.nlm.nih.gov/tools/primer-blast](../../../../../AppData/Local/Microsoft/Windows/Temporary%20Internet%20Files/Content.Outlook/AppData/Local/Microsoft/Windows/Temporary%20Internet%20Files/Content.Outlook/1RNMM6J4/www.ncbi.nlm.nih.gov/tools/primer-blast)) and checked *in silico* at UCSC ([https://genome.ucsc.edu](https://genome.ucsc.edu/)). HPLC-purified primers were obtained from Eurogenetec. Specificity and sensitivity of the systems were evaluated on 1 ng of human genomic DNA (Promega) by varying the annealing temperature (Tm) from 52°C to 60°C, and amplification cycles were followed by High Resolution Melting (HRM) using Rotor Gene Q (Qiagen), gel electrophoresis analysis on Bioanalyzer 2100 (Agilent), and product sequencing (GATC Biotech). Systems were validated if primer pairs met three criteria: (i) one HRM peak, (ii) fragment size corresponding to the expected product, and (iii) matching the targeted locus sequence after Sanger sequencing. Primer pairs along with an illustration of the experimental validation scheme were summarised in Additional file 6: Figure S4 . For Real-Time PCR, one annealing temperature (56°C) was selected for all repeat-element systems validated, all criteria were validated for all systems at this temperature, and it enabled homogenization of the experiment. The 32 out of 44 primer pairs meeting our acceptance criteria were used to amplify the original RNA samples used for microarray experiments and RNA from 6 additional healthy volunteers similarly, LPS/ET stimulated.

*Real-Time PCR and data analysis*

mRNA expression levels were quantified using RT-qPCR. Total RNA (100ng) was DNAse-treated and reverse-transcribed using QuantiTec Reverse Transcription Kit (Qiagen). Reverse-transcriptase-free reactions were carried out to verify the absence of contaminating genomic DNA using the TaqMan Gene Expression Assay Human 18S system Hs03003631-g1 and TaqMan Universal PCR kit (both ThermoFisher). For repeat-element systems, SYBR green experiments were set up using the Type-it HRM PCR kit in a 20 µL final reaction volume with 0.7 µM primers and a 10-fold cDNA dilution (2 ng RNA equivalent). PCR amplifications were carried out in strip tubes which were closed by caps (all Qiagen). The cDNA amplifications were performed using Rotor Gene Q as follows: a 5-min denaturation step at 95°C, followed by 40 cycles (95°C for 10s, Tm for 30s, 72°C for 10s) and HRM analysis (from 65°C to 95°C, 0,1°C increments every 2s). All reactions were performed in duplicate. Expression of housekeeping genes: *Peptidylprolyl Isomerase B* (PPIB) and *Ribosomal Protein Lateral Stalk Subunit 0* (RPLP0) was monitored for normalisation and other gene expression was investigated using TaqMan Universal PCR Master Mix and TaqMan Gene Expression Assay Human: PPIB (Hs00168719_m1), RPLP0 (Hs00420895_gH), TNFα (Hs00174128_m1), IL10 (Hs009616228m1), (ThermoFisher). The PCR reactions were performed using Rotor Gene Q in strip tubes with a 20 µL final reaction, primers and mix concentration and amplification program were determined according to the manufacturer’s instructions (Thermofisher). The fold change (FC) was determined using the 2^-ΔΔCt^ method. The first ΔCT is the difference in threshold cycle between the target and the geometric mean of the PPIB and RPLP0 genes; ΔΔCT is the difference in ΔCT between the target and the unstimulated reference condition. The final value of the unstimulated condition was arbitrarily set to one, and other values scaled-up in order to provide a final relative differential expression. Statistically significant differences between two conditions are marked (wilkoxon signed rank test. *** p-value < 0.01, **: p-value < 0.05, and *: p-value < 0.1). The use of a less stringent p-value threshold (0.1) is explained by the low number of samples and the inter-individual variability observed, reducing statistical power to detect effect when there actually is.

1. Huber W, Carey VJ, Gentleman R, Anders S: **Orchestrating high-throughput genomic analysis with Bioconductor**. 2015, **12**(2):115-121.

2. Wilson CL, Miller CJ: **Simpleaffy: a BioConductor package for Affymetrix Quality Control and data analysis**. *Bioinformatics* 2005, **21**(18):3683-3685.

3. Kauffmann A, Gentleman R, Huber W: **arrayQualityMetrics--a bioconductor package for quality assessment of microarray data**. *Bioinformatics* 2009, **25**(3):415-416.

4. Irizarry RA, Hobbs B, Collin F, Beazer-Barclay YD, Antonellis KJ, Scherf U, Speed TP: **Exploration, normalization, and summaries of high density oligonucleotide array probe level data**. *Biostatistics (Oxford, England)* 2003, **4**(2):249-264.

5. Johnson WE, Li C, Rabinovic A: **Adjusting batch effects in microarray expression data using empirical Bayes methods**. *Biostatistics (Oxford, England)* 2007, **8**(1):118-127.

6. Perot P, Mullins CS, Naville M, Bressan C, Huhns M, Gock M, Kuhn F, Volff JN, Trillet-Lenoir V, Linnebacher M *et al*: **Expression of young HERV-H loci in the course of colorectal carcinoma and correlation with molecular subtypes**. *Oncotarget* 2015, **6**(37):40095-40111.
